# Normative data for instrumented posturography: a systematic review and meta-analysis

**DOI:** 10.3389/fnhum.2024.1498107

**Published:** 2024-12-18

**Authors:** Angela Julienne, Evi Verbecque, Stéphane Besnard

**Affiliations:** ^1^[UR 7480 VERTEX (Vertige Extrême)], University of Caen Normandy, Caen, France; ^2^Reval (Rehabilitation Research Center), Faculty of Rehabilitation Sciences, Diepenbeek, Belgium

**Keywords:** normative data, postural control, systematic review, meta-analysis, Posturography test

## Abstract

Postural control is a multisensory adaptive system performing predictive (anticipatory) and/or reactive (compensatory) actions, with varying degrees of accuracy, to maintain balance in a changing environmental context. Common instrumentation to evaluate balance includes static and dynamic force platforms; added sway-referenced perturbations on the dynamic platform constitute its main advantage. Clinical applications notwithstanding, normative data are needed for interpretation in clinical settings. Posturography norms are used to compare a reference group (healthy individuals) and a specific patient population. This work, to the best of our knowledge, represents the first attempt to synthesize the literature on normative data for computerized posturography using a combined mixed method. The search strategy resulted in the retrieval of 1,244 articles from PubMed, Web of Science, and Science Direct. After deduplication, 689 articles were screened based on title and abstract. One hundred and seven articles met the criteria after the first screening. In-depth, full-text screening resulted in the inclusion of 44 studies for the systematic review and 17 studies for the meta-analyses. The main findings of the systematic review are (1) extensive heterogeneity was found in methodological characteristics, (2) there was insufficient risk of bias mitigation, (3) the majority of tasks evaluated less than four components of the systems framework for postural control (SFPC), and (4) studies mostly used distance domain sway parameters and did not report the influence of other variables on postural sway. Based on the multilevel meta-analyses, females appeared to outperform males in eyes closed (EC) conditions significantly. Based on the network meta-analyses, we found that younger children swayed more than those aged between 8 and 14 years both in eyes open (EO) conditions and EC conditions significantly. The results also revealed a significant difference in sway between individuals of age range between 50 and 79 years old and younger individuals, with more instability observed in older participants both in EO conditions and in EC conditions. Thus, future studies need to ensure that enough information about participants is provided. Standardization of experimental conditions and sway parameters harmonization are still needed to ensure high-quality assessment (QA). Finally, evidence-based postural impairment management requires both age- and sex-related normative data.

**Systematic review registration:**https://www.crd.york.ac.uk/prospero/display_record.php?ID=CRD42023378144, identifier PROSPERO 2023 CRD42023378144.

## 1 Introduction

Balance arises from a multisensory adaptive system performing predictive (anticipatory) and/or reactive (compensatory) actions, with varying degrees of accuracy, to impact balance in changing environmental contexts (Pollock et al., 2000). Numerous techniques and methods are employed to evaluate postural control at a functional and neurophysiological level, in static and dynamic conditions (Paillard and Noé, 2015). Basic non-instrumented tests, such as the Timed Up and Go test (Podsiadlo et al., 1991), the Berg Balance Scale (Berg et al., 1992), and the Tinetti Balance Scale (Tinetti, 1986), among others, are well known and widely used by clinicians. However, these tests give a broad overview of the functional state. In contrast, instrumented tests offer the possibility to carry out in depth quality of movement assessment under different conditions and difficulty levels. Instrumented tests evaluating balance are carried out through computerized force platforms calculating the displacements of the center of pressure in static and dynamic conditions (Bizzo et al., 1985). When standing and walking, the point of application of the ground reaction forces can be measured as the center of the pressure signal using linear and non-linear variables (Quijoux et al., 2021).

Many instrumented devices are available on the market for clinical and research use. Common instrumentation includes static and dynamic force platforms. Both platforms can assess postural control; the added sway-referenced perturbations on the dynamic platform constitute its main advantage. These perturbations allow the evaluation of sensory integration, reweighting, and the use of sensory strategies while standing and walking. The most used static platforms are laboratory-grade force plates manufactured by AMTI (AMTI, Watertown, MA, United States) and Kistler (Kistler, Winterthur, Switzerland). Other low-cost alternatives also exist, such as the Wii Balance Board (WBB; Nintendo, Kyoto, Japan), which is considered a reliable and valid tool [for review, see Clark et al. (2018)]. In general, studies investigating the reliability and validity of the Wii Balance Board mostly used path length as a sway parameter to evaluate healthy participants. Numerous studies have used conventional force platforms manufactured by Neurocom International, Inc., Clackamas, OR, USA. EquiTest^®^ device—a Computerized Dynamic Posturography (CDP) system—is considered a gold standard instrument. The concept of CDP—an apparatus and method for sensory integration and motor coordination analysis—was developed and patented by Nashner in 1988. The sensory and motor components of human postural control can be assessed by different standardized tests (Sensory Organization Test [SOT], Motor Coordination Test [MCT], Adaptation Test [ADT], and Limits of Stability) for which manufacturers have commercially published normative data. For these tests, balance is measured using equilibrium and composite scores as well as sway energy and maximal voluntary movement parameters (e.g., directional control and reaction time).

Furthermore, instrumented posturographic tools have been used to investigate balance deficits, especially in populations at higher risk of falls (Achour Lebib et al., 2006; Bloem et al., 2003). For example, the CDP’s role has been widely studied in neurotology (Black et al., 1983; Horak et al., 1990; Nashner et al., 1994; Nashner and Peters, 1990; Wall et al., 1983). Increased instability has been reported in patients with vestibular disorders. SOT results have shown that patients exhibit impaired performances during conditions 5 (fixed visual surround and sway-referenced platform) and 6 (sway-referenced visual surround and platform), which is indicative of a vestibular deficit affecting the postural functioning of the individual (Black et al., 1988). Furthermore, the results of dynamic posturography in a broad spectrum of patients with peripheral and central vestibular disorders revealed varying degrees of functional capacity within each group. Psychometric properties for dynamic posturography have been mainly studied in this specific population (Di Fabio, 1995, 1996; Hamid et al., 1991). Analogous to CDP, computerized static posturography, using a foam-supported platform as a perturbation, offers a low-cost and portable alternative. Thus, the Clinical Test of Sensory Interaction on Balance (CTSIB) can evaluate balance in eyes open (EO) and eyes closed (EC) conditions with/without foam. Increased sway was observed in patients compared to controls during foam posturography, particularly with EC, measured using amplitude and velocity (Baloh et al., 1998) as well as velocity, area, and Romberg’s ratio (Fujimoto et al., 2009). In the CTSIB, integrated with virtual reality, instability was condition dependent—patients with vestibular disorders sway more during more challenging sensory conditions (i.e., foam surface, EC, and optokinetic stimulus) (Macedo et al., 2015).

Clinical applications notwithstanding, normative data are needed for interpretation in clinical settings. Normative data (SpringerReference) are observational data summarizing and describing a population’s characteristics at a specific time. Posturography norms are used to compare a reference group (healthy individuals) and a specific patient population. Thus, variations between patients and healthy individuals can be observed and interpreted in a clinical setting to determine pathological profiles. Descriptive information (e.g., total sample size, age groups, and sex) and statistics (e.g., median; other summaries of distribution; indicators of central tendency and dispersion; and standard scores) are needed to report such datasets. Some studies have focused on establishing reference values for CDP, mostly using the NeuroCom EquiTest (NeuroCom International, Inc), for which normative data are already available. Normative data have also been published by the BTrackS Balance Plate (Balance Tracking systems) parent company for static computerized posturography.

However, one significant limitation has been noted: the lack of stratification and socio-demographic information. Many studies have demonstrated that sex and age influence postural control (Farenc et al., 2003; Hageman et al., 1995; Ionescu et al., 2005). For static computerized posturography, a systematic review of postural sway in children revealed that age stratification was arbitrary in majority of studies, leading to conflicting results concerning postural control development (Verbecque et al., 2016b). Furthermore, results showed that stability increased with age, and children swayed more when visual input (in EC condition) was removed. These results have also been observed in adults. Other variables such as anthropometric characteristics (Alonso et al., 2012; Chiari et al., 2002), feet positioning (Gibbons et al., 2019; Kollegger et al., 1989), and physical activity (García-Soidán et al., 2020; Gauchard, 2003; Lelard and Ahmaidi, 2015) can also affect balance. Thus, lack of stratification and reporting might lead to misinterpretation of patient postural performance in clinical practice and healthy individuals undergoing behavioral and balance assessments. Therefore, there is room for improvement, and this work aspires to complement previous studies.

The purpose of this study is to systematically review the reported normative data, assessed by computerized posturography, in a healthy population and to discuss the strengths and limitations of these norms to outline future perspectives and needs. Overall, we aimed to identify methodological characteristics of computerized posturography assessment in this systematic review and investigate the impact of age and sex in the meta-analysis.

## 2 Methods

### 2.1 Information sources, search strategy, and inclusion criteria

This systematic review and meta-analysis included studies that provide normative data assessed using instrumented platforms in healthy populations (Registration: CRD42023378144). It was conducted in accordance with the Preferred Reporting Items for Systematic Review and Meta-Analysis (PRISMA) statement. A comprehensive literature search was performed in PubMed, Web of Science, and Science Direct electronic database platforms. A search string was used with keywords (1) “normative data”, (2) “posturography”, (3) “instrumented platform” combined using the Boolean operators “OR” and “AND” for interaction among sets of keywords. The searches were conducted in May 2022 and updated in January 2023. The studies were screened based on *a priori* defined inclusion and exclusion criteria using Rayyan, a web-based application (Ouzzani et al., 2016). The literature search was limited to articles published between 1 January 1980 and 1 January 2022. Computerized posturography was created in 1980 (Nashner, 1982). Therefore, only studies published as of this date were included in this review. In each article, the use of instrumented platforms as evaluation tools was sought. Reference lists and citations of the included articles were manually screened to identify additional studies of interest. The literature search was limited to full-text articles that were written in English and French. The articles meeting the criteria based on their titles and abstracts were included, and their full-text versions were then extracted. AJ and EV performed screening. Full-text articles were checked if information in the title and abstract was insufficient to determine eligibility.

### 2.2 Selection criteria and strategy

To select relevant literature, the following selection criteria were applied:

- Healthy participants were a combination of men and women (absence of any other impairments that can influence balance). The normative data had to be representative of the average population of healthy people. However, studies that included athletes, soldiers, and one of the sexes were exclusively considered relevant to the research question. A minimum sample size of 30 participants was considered an adequate representative sample.
- Instrumented equipment during static or dynamic bipedal balance measurement had to be included. Citations were excluded when data were collected during gait and functional measures (e.g., Timed Up and Go, climbing stairs, 6-min walk test, Y-Balance Test, Star Excursion Balance Test, treadmill walking or during running, turning, stepping tasks lateral, forward, backward, etc.). Balance assessment through mobile devices was excluded as well.
- Original research including full-length articles that were written in English or French. Reviews, meta-analyses, case–control studies/series, conference proceedings, abstract only, books/book chapters, letters to the editor, study protocols, pilot studies, editorials, or opinion pieces were excluded.
- Studies that had explicitly reported normative data were included.

### 2.3 Data extraction and study quality assessment

Data extraction was *a priori* defined using a codebook which included the following information: (1) Study characteristics (authors, year of publication, etc.); (2) methodological details, such as population and measurement characteristics, tasks, and sway parameters; and (3) equations used and psychometric properties (if provided). Authors were contacted to obtain any unavailable data. At a systematic review level, we evaluated the stratification of age and sex and the significance of other factors tested in each study. These are expressed as significant (S), not significant (NS), and not reported (NP).

Studies used a combination of different support and visual conditions, which can be divided into distinct groups and subgroups. In this review, balance tasks were described according to measurement and movement control type as well as sensory perturbations based on a classification process used by Herssens et al. (2020): without sensory perturbations, with proprioceptive perturbations (foam, moving platform and/or foot placement), with visual perturbations (deprivation and/or altered surround), with vestibular perturbations (head movements), and with multiple sensory perturbations. A distinction between measurement and movement control types was made to describe performance in the present review. External conditions determine measurement type during balance assessment. It is classified as dynamic when tasks are executed in altered visual and/or tactile environments and static when tasks are executed in stable visual and/or tactile environments. Movement control type is considered activity-based conditions represented as tasks consisting of quiet standing (static) and voluntary body movements (dynamic). In addition, we used the Systems Framework of Postural Control (Sibley et al., 2015) to determine the nine components of balance (functional stability limits, underlying motor systems, static stability, verticality, reactive postural control, anticipatory postural control, dynamic stability, sensory integration, cognitive influences) which were assessed in the different tasks.

To the best of our knowledge, no quality assessment (QA) tool exists for reference values. However, we adapted the quality assessment tool for diagnostic accuracy studies (QUADAS) scale reported? (Whiting et al., 2003, 2006) and selected the following relevant questions (1, 2, 5, 9, 12, and 13) based on our study objectives: (1) Was there adequate sampling of normative data? (2) Were selection criteria clearly described? (5) Was a motor scale (i.e., other than posturographic measurement) used as part of the test battery? (9) Was the description of instruction and position/setup for the patient, type of equipment, and the outcomes at least reported? (12) Was the age and sex of a sample at least available? (13) Were drop-outs and falls collected (e.g., was it reported that a portion of the participants fell) and reported? Answers were scored in “Yes” or “No” forms, and missing or ambiguous details were scored as “Unclear.” The total score has not been defined for the QUADAS tool. Therefore, studies were considered high quality (low risk of bias) if four or more items out of six items were scored “Yes.” AJ and EV independently performed a QA for each article, and each assessor was blind to the score the other gave. Any disagreement over the final score for each article was discussed until consensus.

**Figure 1:**
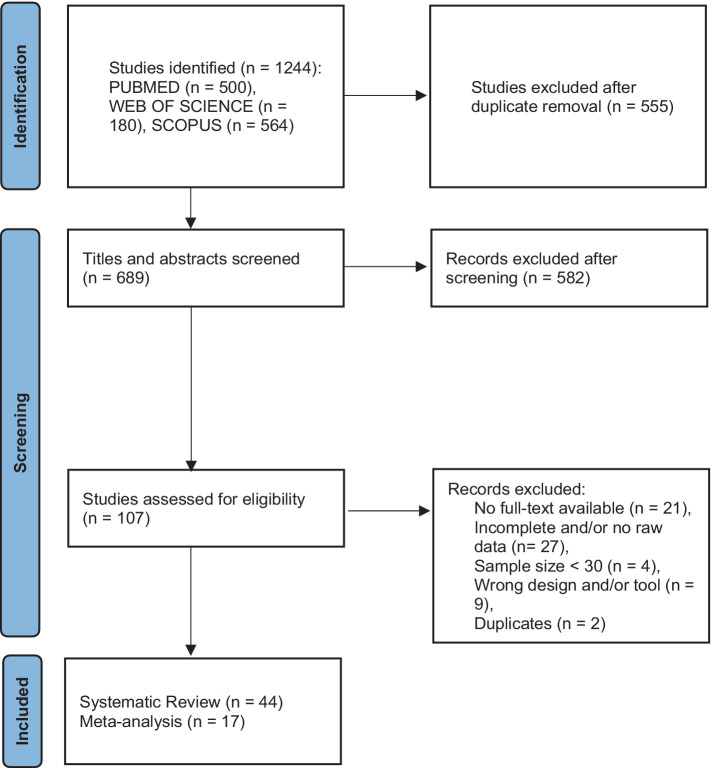
Flowchart for the systematic review and meta-analysis.

Studies providing summary statistics were included in the meta-analysis. Quantitative analysis was done after extraction by categorizing and grouping according to population type (adults, children, and both) and sex distribution (females and males) as well as posturography type (static and dynamic), tasks, and sway parameters. We performed a meta-analysis on sway parameters used by at least three publications in similar measurement conditions. Priority was given to age and/or sex-stratified reference values to mitigate inter study variability. When possible, participants’ mean age and groups were converted and pooled according to decade for adults and biological ages for children to reduce heterogeneity. We used a broad age categorization to ensure enough studies were included in the quantitative analysis to allow for meaningful interpretation. Using to the Cochrane Handbook (Higgins et al., 2020), subgroups were combined into a single group to compare males and females separately and to compare specific age groups whenever possible. When possible, specific terms were attributed to sway parameters based on the definitions in the literature (Prieto et al., 1996) to aggregate data and comparison for meta-analysis purposes. Homogeneity should have been particularly present across age/sex stratification and test conditions. Additionally, quantitative analysis was not conducted when an insufficient number of identified studies were available per task condition (EO and EC, foam, etc.) (*n* > 5). We found that 38 studies used sway parameters used by at least three publications. After further inspection, we removed studies that had no age and/or sex comparisons, insufficient statistics, or were owned by a company (*n* = 21). Instead, these studies were summarized narratively (see Figure 1). The majority of the excluded studies had reported a decrease in sway with increasing age for children whereas an increase was observed for aging adults. Sensory perturbations tended to increase instability, and overall, females outperformed males. Studies assessing children did not investigate the influence of other factors.

### 2.4 Statistical analysis

Statistical analyses were performed in R version 4.2.3^1^ using the packages, metafor (Viechtbauer, 2010), clubSandwich (Pustejovsky, 2024), and netmeta (Balduzzi et al., 2023). Age and sex were analyzed in each sensory condition using different models with effect sizes nested within outcomes, and the latter within studies. We undertook meta-analyses if participants, interventions, outcomes, and comparisons were sufficiently similar. Potential modifiers included population characteristics and intervention details, which are considered sources of heterogeneity.

#### 2.4.1 Measures of group effect

The standardized mean difference was calculated to pool effect sizes to quantify the difference in performance between age and sex groups. For measures where a higher score represented better performance (e.g., equilibrium scores in EO and EC), the direction of the scale was reversed by multiplying by −1 to ensure interpretability across studies. Furthermore, we accounted for a hierarchical dependence structure as multiple effect sizes were extracted from the same studies, experiments, and outcomes across samples for the multilevel meta-analysis.

#### 2.4.2 Outliers and influential cases

Standardized deleted residuals and Cook’s distance were used to identify outliers and influential studies. Residuals larger than ±1.96 indicated that studies did not fit the model and thus represented outliers. Moreover, influential cases were removed when Cook’s distance was over 4/*n*, where *n* is the total number of data points.

#### 2.4.3 Assessment of heterogeneity

In the multilevel meta-analysis, we assessed heterogeneity using the I2 statistic to quantify the percentage of variation attributable to each level – study, outcome, and effect size (low: 25%, moderate: 50%, high: 75%; Higgins et al., 2020). In the network meta-analysis, global and local approaches were used to assess heterogeneity (i.e., variation in effect modifiers within comparisons) and inconsistency (i.e., imbalance in effect modifiers between comparisons). Specifically, we fitted Cochran’s Q (χ^2^) statistic decomposition to evaluate the contribution of each design to the heterogeneity “within designs” and consistency “between designs.” Moreover, a design by treatment (in our case, age groups) interaction model was also fitted for the global approach. Local inconsistency between direct and indirect evidence was evaluated using the Separate Indirect from Direct Evidence (SIDE) method (Dias et al., 2010). To explore sources of heterogeneity, a meta-regression was conducted.

#### 2.4.4 Assessment of publication bias

The potential publication bias was addressed by estimating the funnel plot asymmetry and was tested via Egger’s regression when more than 10 studies were included.

#### 2.4.5 Multilevel meta-analysis using robust variance estimation

We conducted a multilevel meta-analysis using robust variance estimation (RVE) to synthesize studies comparing females and males. To account for the complexity of the data structure, an RVE method can be used to model dependencies between effect sizes and their correlated sampling errors. To obtain robust confidence intervals and *p*-values, the Sandwich estimator (package clubSandwich) was used in combination with the model. For smaller numbers of included studies, valid analysis results are ensured by an adjustment matrix based on the bias-reduced linearization CR2 method. An assumption of the degree of correlation was estimated at *r* = 0.6. The RVE model provides a valid overall average effect size even if the correlation assumption is inaccurate. Several sensitivity analyses for varying values of r were nonetheless conducted.

#### 2.4.6 Frequentist network meta-analysis: multiple interventions

We conducted a network meta-analysis (NMA) to estimate the relative effects for all possible comparisons between different age groups. The frequentist random-effects network meta-analysis considers direct and indirect evidence, also called mixed evidence (i.e., direct—studies comparing A vs. B; indirect—studies comparing A vs. C) (Rouse et al., 2017). Thus, this method estimates the relative effects of comparisons between groups via one or more intermediate comparators or direct ones. The network structure was inspected, and subnetworks (i.e., not fully connected networks) were analyzed separately. Furthermore, studies with missing or inconsistent group estimates and variances were also excluded from the network meta-analysis. The age groups (i.e., interventions) were ranked using the surface under the cumulative ranking curve (SUCRA) method (Salanti, 2011) to account for the entire distribution of the relative effects. This score is expressed as a percentage, with the best ranking corresponding to 100%. This analysis allowed us to include all possible direct and indirect comparisons of different age groups and determine their rankings. Furthermore, we assessed the certainty of the evidence for each comparison using Confidence in Network Meta-Analysis (CINeMA), a web application used to evaluate the confidence of the evidence estimates from network meta-analysis. It is based on the following six domains: within-study bias (risk of bias), reporting bias (publication bias), indirectness (i.e., relevance to the research question), imprecision (comparing the range of effects with the range of equivalence), and incoherence (disagreement between direct and indirect evidence). We excluded two studies (Libardoni et al., 2018; Micarelli et al., 2020) from the meta-analysis in EO and EC conditions due to inconsistent group estimates and variances.

## 3 Results

### 3.1 Information sources and search strategy

The search strategy resulted in the retrieval of 1,244 articles from PubMed, Web of Science, and Science Direct, on 9 May 2022 and a second search on 31 December 2022. After deduplication, 689 articles were screened based on title and abstract. One hundred and seven articles met the criteria after the first screening. In-depth, full-text screening resulted in the inclusion of 44 studies for the systematic review. An overview of the study design is summarized in the PRISMA flowchart (Figure 1).

### 3.2 Descriptive synthesis: risk of bias assessment and methodological characteristics

**Figure 2:**
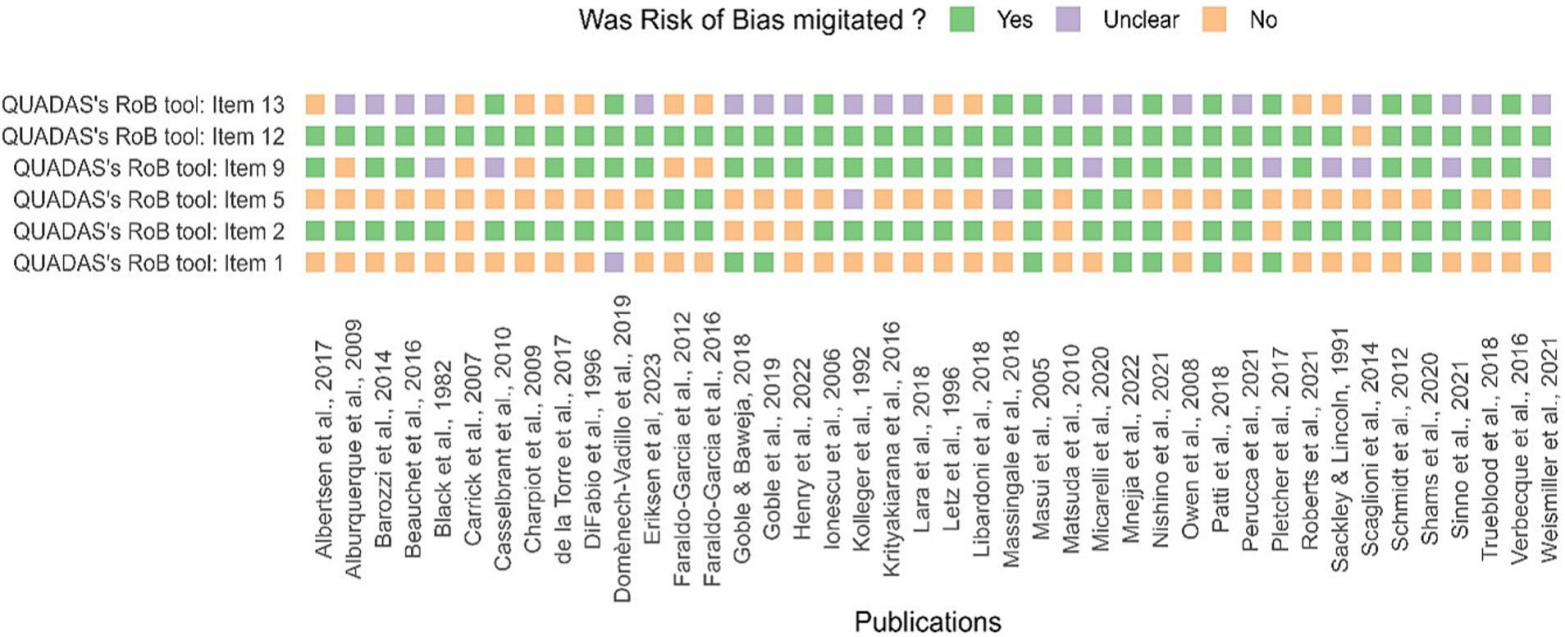
Assessment of study quality (QA) and risk of bias. The risk of bias, using QUADAS’s tool, for each study, is included in the systematic review. Answers were scored “Yes” or “No,” and missing or ambiguous details were scored as “Unclear.” Unreported details were scored as an ‘unclear’ risk of bias.

The risk of bias assessment reveals extensive variation in methodological characteristics (see Supplementary Table S1). Ten studies had high-quality assessment and took measures to reduce risk of bias (Figure 2). The majority of the problematic aspects were in questions 1, 5, and 13 of the adapted QUADAS.

#### 3.2.1 Participants

The general characteristics of the included studies are presented in Table 1. The sample size of publications included in the systematic review ranged between 30 and 16,357 participants. In total, 42,408 participants (12,043 women, 13,761 males) were included and represented mostly regular individuals (77%). Children and adults combined were primarily represented in the sample (48%). Specific cohorts were included in a few studies, such as athletes (11%), military (6.8%), and mixed (4.5%). The majority of the studies were conducted in Europe (41%) and North America (36%). The study population represented a large range of ages and participants comprising of children, adolescents, and adults. Age and sex were stratified, respectively, in 64 and 45% of the publications. Regarding age stratification, majority of the studies did not provide age groups (34%) (Albertsen et al., 2017; Beauchet et al., 2016; Carrick et al., 2007; de la Torre et al., 2017; Goble et al., 2019a; Henry et al., 2022; Krityakiarana and Jongkamonwiwat, 2016; Lara et al., 2018; Letz et al., 1996; Matsuda et al., 2010; Owen et al., 1998; Pletcher et al., 2017; Scaglioni-Solano and Aragón-Vargas, 2014; Schmidt et al., 2012; Weismiller et al., 2021); some reported values per biological age (18%) (Barozzi et al., 2014; Casselbrant et al., 2010; di Fabio and Foudriat, 1996; Ionescu et al., 2005; Libardoni et al., 2018; Micarelli et al., 2020; Mnejja et al., 2022; Verbecque et al., 2016a) and per decade (16%) (Black et al., 1982; Eriksen and Hougaard, 2023; Faraldo-García et al., 2012, 2016; Goble and Baweja, 2018b; Sackley and Lincoln, 1991; Trueblood et al., 2018), or academic level associated with school year (11%) (Charpiot et al., 2010; Goble et al., 2019b; Massingale et al., 2018; Shams et al., 2020; Sinno et al., 2021). Others used an unconventional stratification method (Alburquerque-Sendín et al., 2009; Domènech-Vadillo et al., 2019; Goble and Baweja, 2018a; Kollegger et al., 1992; Masui et al., 2005; Nishino et al., 2021; Patti et al., 2018; Perucca et al., 2021; Roberts et al., 2021). Several studies did not provide explicit reasons for omitting age stratification. However, a few studies decided to pool data as they did not observe age-based differences. We speculate that sample size constraints and the complexities of recruiting older participants may have been contributing factors, as suggested by a few studies.

**Table 1:** General characteristics of included studies.

Characteristic	*N* = 44^1^
Population group	
Adults	14 / 44 (31.8%)
Both	21 / 44 (47.7%)
Children	9 / 44 (20.4%)
Individuals’ category within each population	
Athletic	5 / 44 (11%)
Military	3 / 44 (6.8%)
Mixed	2 / 44 (4.5%)
Regular	34 / 44 (77%)
Region	
Africa	1 / 44 (2.3%)
Asia	5 / 44 (11%)
Central/South America	4 / 44 (9.1%)
Europe	18 / 44 (41%)
North America	16 / 44 (36%)
Sample size	30–16,357; 42,408
Females	0–4,292; 12,043
Not reported	3
Males	0–6,624; 13,761
Not reported	3
Stratified by age	
No	16 / 44 (36%)
Yes	28 / 44 (64%)
How was age stratified?	
Academic	5 / 44 (11%)
Biological	8 / 44 (18%)
Decade	7 / 44 (16%)
None	15 / 44 (34%)
Unconventional	9 / 44 (20%)
Stratified by gender	
No	24 / 44 (55%)
Yes	20 / 44 (45%)
Post-urographic assessment type	
Dynamic	21 / 44 (48%)
Static	23 / 44 (52%)
Movement control type	
Dynamic	4 / 44 (9.1%)
Static	40 / 44 (91%)
Sensory perturbations used in tasks	
Multiple	32 / 44 (72.7%)
Proprioceptive	3 / 44 (6.8%)
Visual	8 / 44 (18.1%)
Without	5 / 44 (11.3%)
Were equations for calculating sway parameters provided?	
No	32 / 44 (73%)
Yes	12 / 44 (27%)
Were psychometric properties reported?	
No	35 / 44 (80%)
Yes	9 / 44 (20%)

1 n / N (%); Range; Sum.

#### 3.2.2 Tasks and sway parameters

**Figure 3:**
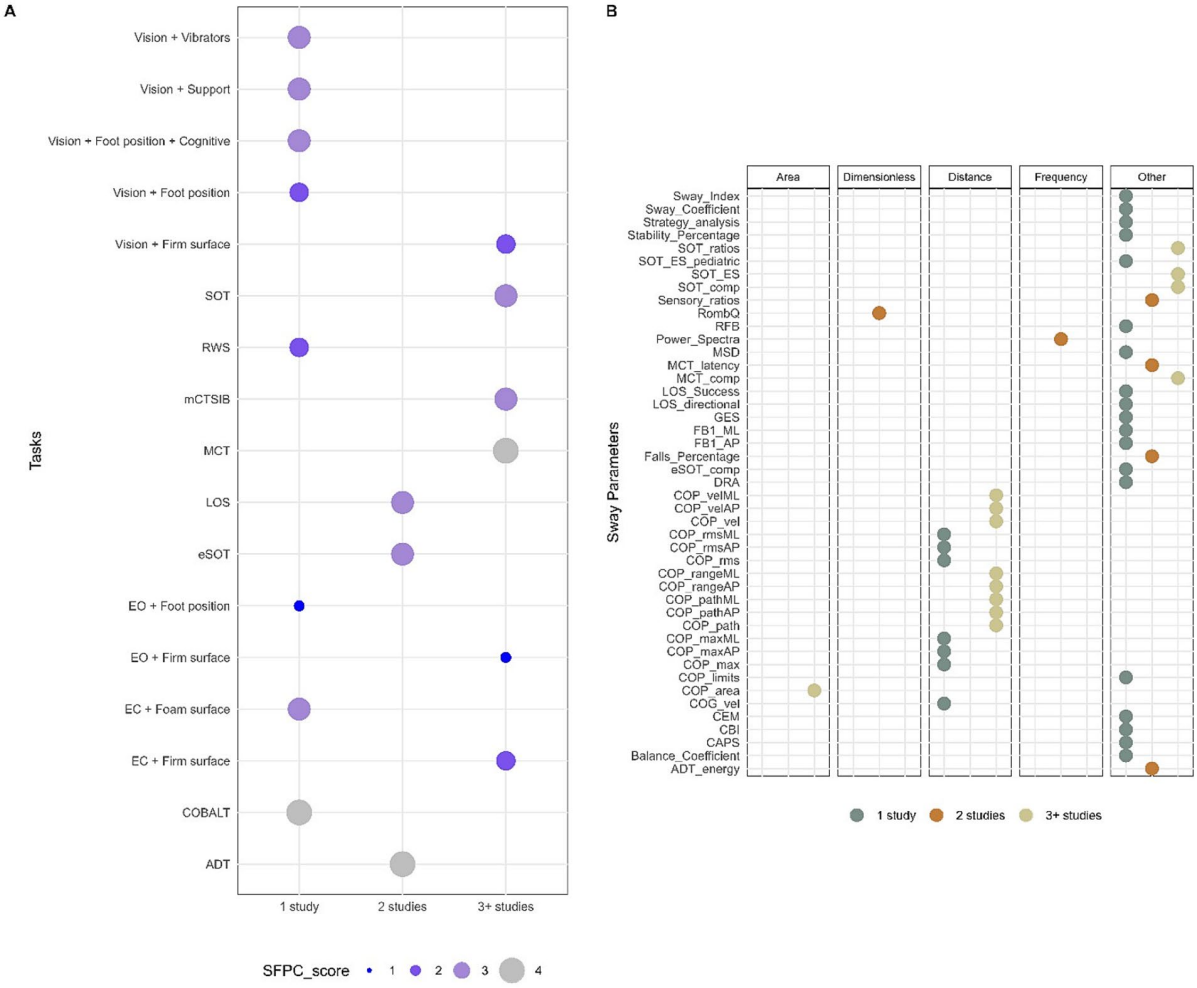
Methodological characteristics for tasks and sway parameters. **(A)** Tasks reported in n ≥ 3 number of studies based on the Systems Framework of Postural Control components evaluated in each task. **(B)** Sway parameters used in *n* ≥ 3 number of studies and grouped by domain.

The included studies investigated postural sway using a variety of tasks. Measurement conditions varied largely regarding arm and foot position, use of the visual target, the number and duration of trials and sequence of the conditions. Twenty-three studies used static measurement, whereas static movement control was described in 40 studies. When possible, tasks were converted into standardized tests commonly used in clinical practice based on specified conditions. If a task did not correspond to a known test, we coded it according to precise sensory conditions. Overall, 17 different tasks were used. The majority of studies used multiple sensory perturbations (70%) and did not report psychometric properties (80%). Using the Systems Framework of Postural Control, components of postural control measured in each task were determined (Figure 3A). Three out of 17 tasks evaluated the majority of the components of the systems framework for postural control (SFPC) (4 items); only one task was used in more than three studies, the Motor Control Test (MCT). Other mostly used tasks were the Modified Clinical Test of Sensory Interaction in Balance (mCTSIB) and the SOT, which evaluated three components of the SFPC (static stability, underlying motor systems, and sensory integration) as well as Vision + FIRM (visual perturbation with firm surface) and EC + FIRM (EC with firm surface) which evaluated two components (static stability and sensory integration). Finally, EO + FIRM (EO with firm surface) was also used and evaluated one component, static stability. The four SFPC components evaluated in the MCT were static stability, underlying motor systems, reactive postural control, and sensory integration.

A large variety of parameters for postural sway led to semantic heterogeneity in the reported data. Therefore, whenever possible, specific terms were attributed to sway parameters in this review based on the definitions in the literature to allow comparison between different studies. The publications used 45 different sway parameters to assess balance performance; only a few provided equations for calculating sway parameters (27%). Thirteen sway parameters were used by more than three studies, which were distance domain features: review of center of pressure (COP) area, COP path, and COP velocity in either the anteroposterior (AP) and mediolateral (ML) direction or in a combined plane, COP range in AP and ML direction as well as SOT ratios, SOT equilibrium scores, SOT and MCT composite scores which are non-domain specific features (Figure 3B).

Other variables were also reported as factors influencing postural control (Supplementary Figure S2A). Five studies reported a significant effect of sensory conditions in adults, whereas height mainly was reported as a non-significant variable in children (*n* = 3) and mixed populations (*n* = 4). However, the majority of the studies did not report the influence of other variables on postural sway.

### 3.3 Quantitative analysis

In the multilevel or network meta-analysis, we pooled data from seventeen studies involving 5,194 participants (females: 2,630; males: 2,564). Furthermore, we did not conduct quantitative analysis for Foam conditions and sway-referenced conditions (SOT 3, SOT 4, SOT 5, and SOT 6) as well as for composite score and ratio outcomes as there was insufficient number of studies (*n* < 5).

#### 3.3.1 Effect of sex on postural sway

**Figure 4:**
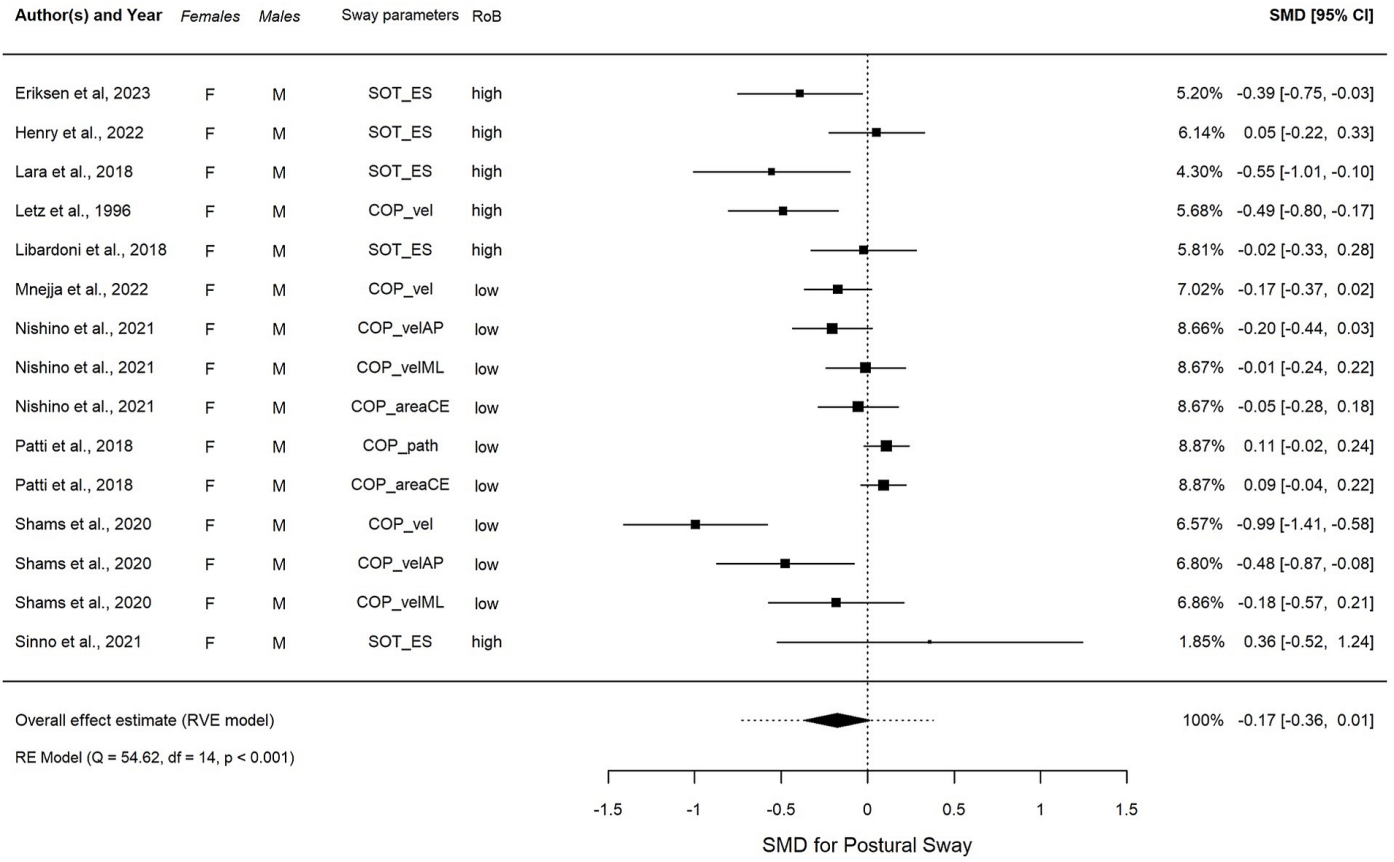
Forest plot visualizing the overall effect in EO, calculated using robust variance estimation (RVE). SMD, standardized mean difference between females and males, CI, confidence interval.

In the EO condition (Figure 4), an overall summary meta-analysis found an average sex difference of sensory modulation disorder (SMD) = −0.17, *t*(7.6) = −2.02, *p* = 0.08, 95% CI: −0.36, 0.01. The overall I2 value indicates that 79.1% was due to heterogeneity, with the variance component accounting for about 28.6% of the total variance at the study level, 25.3% at the outcome level, and 25.3% at the effect size level. The remaining 20.9% was a sampling variance. There were no influential outliers found. Subgroup analyses were conducted since high heterogeneity was detected, and no influential cases were found. Population group, region, and risk of bias were determined *a priori* as potential modifiers. However, none of them could explain explicitly the overall high heterogeneity (Supplementary Table S5). The overall average effect size was not sensitive to our assumption about the correlation between effect size estimates, with estimates varying from −0.187, 95% CI: −0.382, 0.008 assuming 𝜌 = from 0.0 to −0.168, 95% CI: −0.372, 0.036 assuming 𝜌 = 0.9. The total variation in true effect sizes was similarly insensitive, with a total standard deviation (SD) ranging from 0.220 to 0.255. However, individual variance component estimates were more sensitive to the assumed 𝜌 (see Supplementary Table S4A).

**Figure 5:**
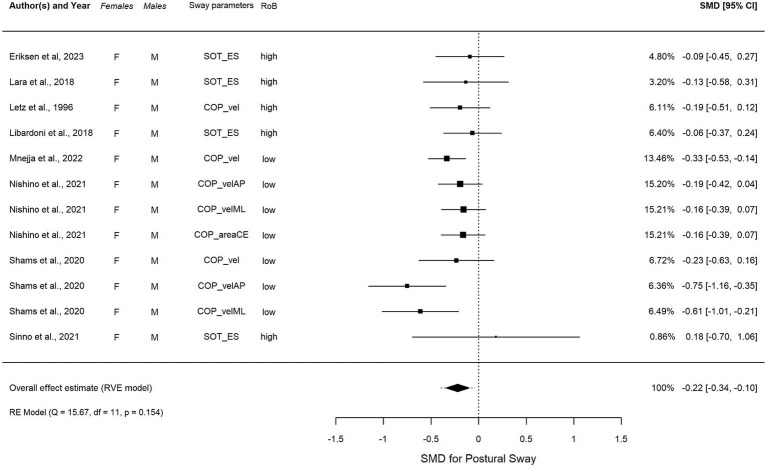
**(A–D)** Forest plot visualizing the overall effect in EC, calculated using robust variance estimation (RVE). SMD, standardized mean difference between females and males; CI, confidence interval.

In EC condition (Figure 5), an overall summary meta-analysis found an estimated overall significant SMD = −0.22, *t*(4.6) = −4.20, *p* = 0.01, 95% CI (−0.34, −0.10), with non-significant low heterogeneity (I2 = 11.6%) after removing one outlier. The overall average effect size was not sensitive to our assumption about the correlation between effect size estimates, with estimates varying from −0.233, 95% CI: −0.396, −0.069 assuming 𝜌 = from 0.0 to −0.211, 95% CI: −0.347, −0.076 assuming 𝜌 = 0.9. The estimated total variation in true effect sizes was similarly insensitive, with a total SD ranging from 0.11 to 0.141. However, individual variance component estimates were more sensitive to the assumed 𝜌 (see Supplementary Table S4B).

#### 3.3.2 Effect of age on postural sway

Network meta-analysis in EO and EC conditions included each of the 10 studies and revealed two subnetworks, which consisted of 22 groups, 358 comparisons, and 8 study designs. The 10 characteristics were relatively similar across groups in each subnetwork, indicating an acceptable transitivity assumption (see Supplementary Table S5). The first and second subnetworks represented adults aged between 20 and 79 years old and children aged between 3 and 14 years old (Supplementary Figure S4). The subnetworks were analyzed separately.

##### 3.3.2.1 Effect of age within adults +20 years

**Figure 6:**
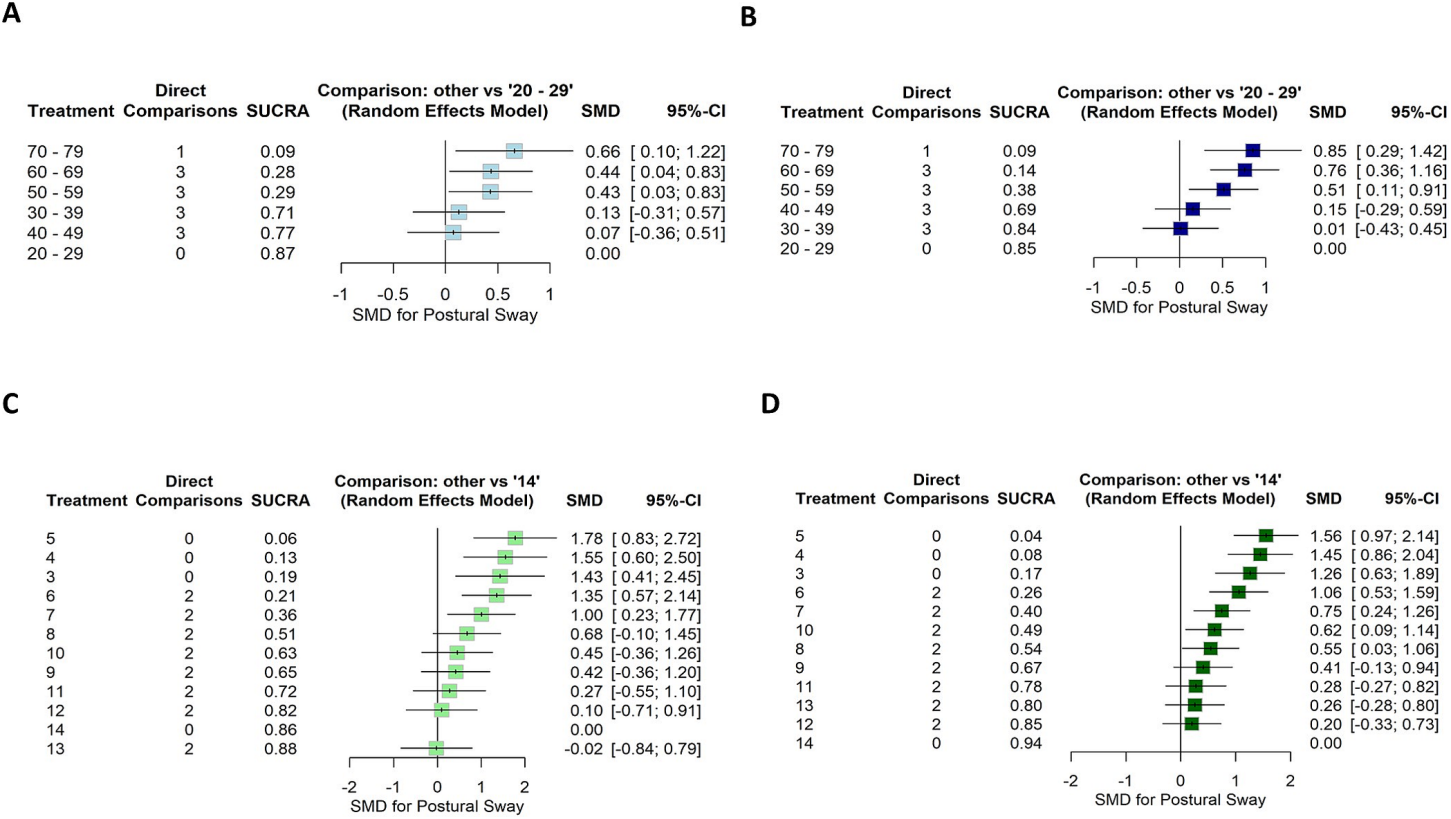
Forest plot showing the SUCRA ranking and effect estimates for EO (left) and EC (right) conditions. **(A,B)** The age group 20–29 years was used as a reference for the first subnetwork analysis. **(C,D)** The age group 14 years was used as a reference for the second subnetwork analysis. SMD, standardized mean difference; CI, confidence intervals.

**Figure 7:**
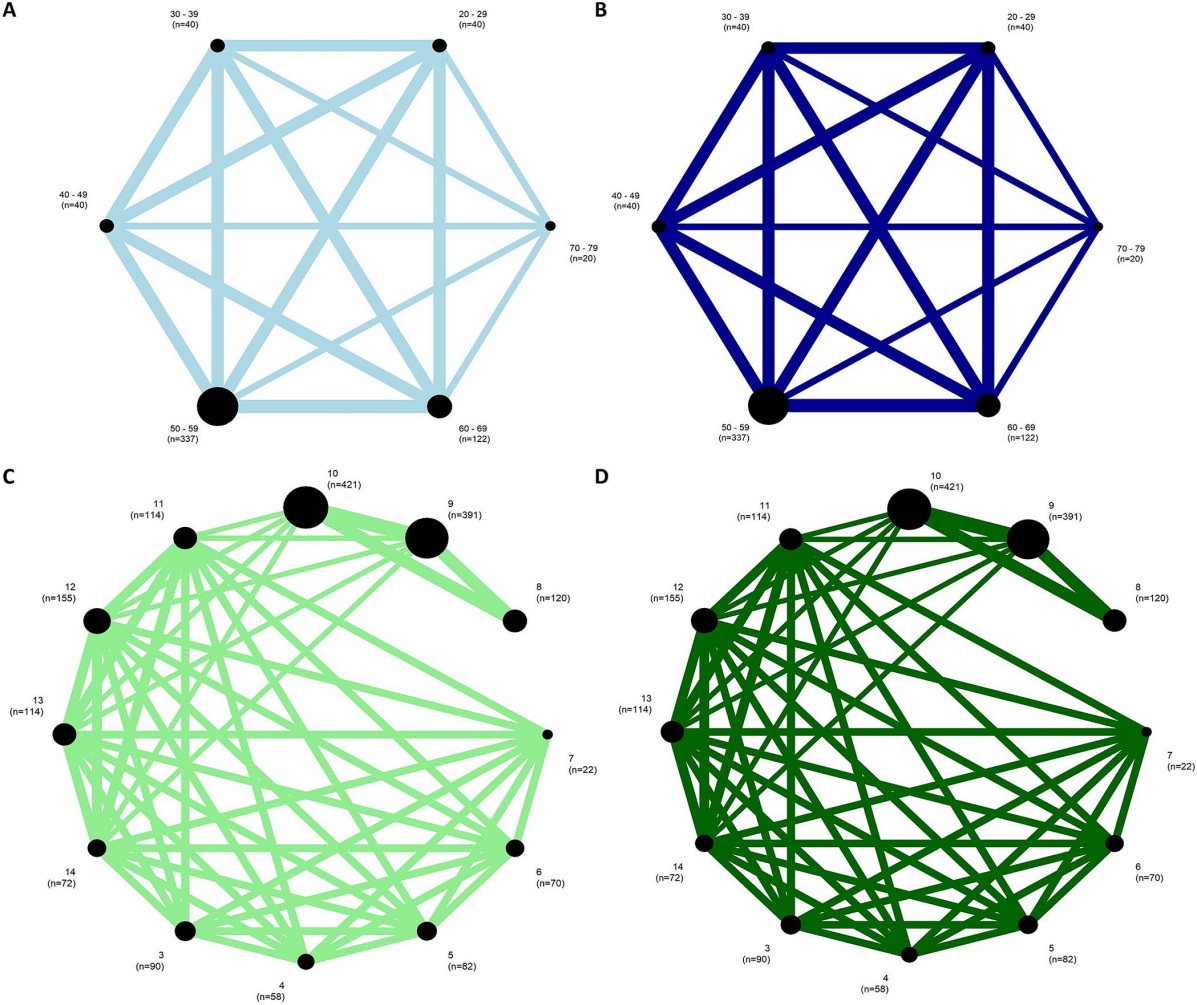
Network plots for EO condition (left) and EC condition (right) of the **(A,B)** first and **(C,D)** the second subnetworks. The width of the lines represents the number of studies comparing each pair of groups. The size of the circle represents the sample size in each arm.

**Figure 8:**
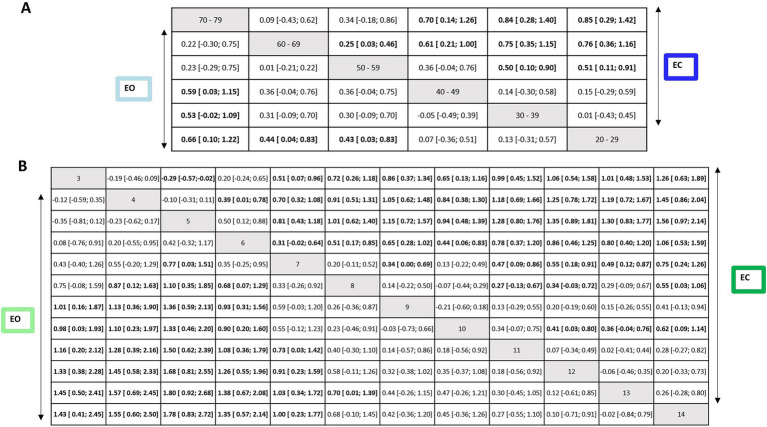
Net league tables for the EO and EC conditions of first subnetwork (upper) and second subnetwork (lower). Relative effect estimates for the contrasts between the different intervention and control arms for the EO condition (lower triangle) and the EC condition (upper triangle). Statistically significant effects are shown in bold letters and highlighted in white cells. SMD, standardized mean difference; CI, confidence intervals.

The first subnetwork analysis, 20–29-year-old participants were used as the reference group. Figures 6A,B shows the first connected subnetworks for both EO and EC conditions. The most frequently examined comparisons were among age groups (in years) 20–29 vs. 30–69 and 30–39 vs. 40–69, and 40–49 vs. 50–69 and 50–59 vs. 60–69. Supplementary Table S6 summarizes the main results of both direct and indirect evidence and the first subnetwork meta-analysis for EO and EC. Figures 7A,B show the treatments’ relative rankings with the group of the age range (in years) 20–29 as the reference group. Figures 7C,D shows the relative rankings with group 14 as the reference. The lowest ranked was the group of the age range (in years) 70–79 (SUCRA: 0.09 for EO and 0.09 for EC) followed by 60–69 (SUCRA: 0.28 for EO and 0.14 for EC) and 50–59 (SUCRA: 0.29 for EO and 0.38 for EC). We present the summary of relative effects in league tables (see top table, Figure 8).

In the EO condition, older groups of the age ranges (in years) 50–59, 60–69, and 70–79 were significantly more instable than the reference group 20–29 (EO: SMD range 0.43–0.66). The groups of the age ranges (in years) 30–39 and 40–49 were more stable than older groups 60–69 (EO: SMD range 0.61–0.75) and 70–79 (EO: SMD range 0.53–0.59).

In EC condition, older groups of the age ranges (in years) 50–59, 60–69, and 70–79 were significantly more instable than the reference group 20–29 (EC: SMD range 0.51–0.85). The groups of the age ranges (in years) 30–39 and 40–49 were more stable than the older group 70–79 (EC: SMD range 0.70–0.84). A statistically significant difference was also found for the age ranges (in years) 50–59 compared to 30–39 (SMD = 0.50, 95% CI: 0.10, 0.90) and 60–69 (SMD = 0.25, 95% CI: 0.03, 0.46); indicating that older individuals swayed more for EC.

The heterogeneity and global inconsistency were low in the first subnetwork (EO: I2 = 0, 95% CI: 0, 62.4%, *χ*^2^ = 8.59, *df* = 9, *p* = 0.47; EC: I2 = 0, 95% CI: 0, 62.4%, *χ*^2^ = 8.41, *df* = 9, *p* = 0.49). Inconsistency between designs appeared to be not an issue in this subnetwork (EO: *p* = 0.7; EC: *p* = 0.6). Supplementary Tables S8A,B summarizes the local inconsistency between direct and indirect evidence using the SIDE method for the EO and EC conditions. The results show that there are 11 pairwise comparisons contributing both direct and indirect evidence, for none of which there is evidence of inconsistency for the EO condition (Supplementary Table S8A). However, a handful of comparisons with inconsistency for EC was observed (*p* < 0.10 refer to Supplementary Table S8B).

##### 3.3.2.2 Effect of age within children ≤14 years

In the second subnetwork analysis, 14-year-old participants were used as the reference group. Figures 6C,D shows the second connected subnetworks for both EO (Figure 5C) and EC (Figure 5D) conditions. The most frequently examined comparisons were between 8 vs. 9, 8 vs. 10, and 9 vs. 10. Supplementary Table S7 summarizes the main results of both direct and indirect evidence and the first subnetwork meta-analysis for EO and EC. Figures 6C,D shows the relative rankings with group 14 as the reference. The lowest ranked was group 5 (SUCRA: 0.06 for EO and 0.04 for EC), followed by 4 (SUCRA: 0.13 for EO and 0.08 for EC), 3 (SUCRA: 0.19 for EO and 0.17 for EC), 6 (SUCRA: 0.21 for EO and 0.26 for EC), and 7 (SUCRA: 0.19 for EO and 0.17 for EC). The summary relative effects from the network meta-analysis are presented in league tables of each subnetwork (see bottom table, Figure 8).

In the EO condition, children aged between 3 and 7 years swayed significantly more than the reference group 14 (EO: SMD range 1.00–1.78). Between 3 and 6 years of age group, children were more instable than older groups: 9 years (EO: SMD range 0.93–1.36), 10 years (EO: SMD range 0.90–1.33), 11 years (EO: SMD range 1.0–1.50), 12 years (EO: SMD range 1.26–1.68), and 13 years (EO: SMD range 1.38–1.80). A statistically significant difference was also found for the group of age 7 years compared to that of 11 years (EO: SMD = 0.73, 95% CI: 0.03, 1.42), 12 years (EO: SMD = 0.91, 95% CI: 0.23, 1.59) and 13 years (EO: SMD = 1.03, 95% CI: 0.34, 1.72). A significant difference was also observed between groups 5 vs. 7 for EO (SMD = 0.77, 95% CI: 0.03; 1.51). For children aged 8 years, a significant difference was reported compared to the younger children for EO (SMD for the age group of 4–6 years range 0.87–1.10) and to group 13 years (SMD = 0.70, 95% CI: 0.01, 1.39). Children aged 5 years swayed significantly more than those aged 7 for EO (SMD = 0.77, 95% CI: 0.03, 1.51).

In EC condition, children aged between 3 and 7 years swayed significantly more than the reference group of age 14 years (EC: SMD range 0.75–1.56). Between 3 and 6 years of age, children were more instable than the older groups of age 9 years (EC: SMD range 0.65–1.15), 10 years (EC: SMD range 0.44–0.94), 11 years (EC: SMD range 0.78–1.28), and 12 years (EC: SMD range 0.86–1.35), and 13 years (EC: SMD range 0.80–1.30). A statistically significant difference was also found for the age group of 7 years compared to 11 years (EC: SMD = 0.47, 95% CI: 0.09, 0.86), 12 years (EC: SMD = 0.55, 95% CI: 0.18, 0.91), and 13 (EC: SMD = 0.49, 95% CI: 0.12, 0.87). A significant difference was also observed between younger children and the age group of 7 years for EC (SMD for groups 3–6 years range 0.31–0.81). A significant difference was reported for children aged 8 years compared to older children (EC SMD for 11–12) range 0.27–0.34 and 14 (EC SMD = 0.55, 95% CI: 0.03, 1.06). Children aged 5 years swayed significantly more than the age group of 3 years for EC (SMD = −0.29, 95% CI: −0.57, −0.02). A significant difference was observed between age groups 7 and 9 years (SMD = 0.34, 95% CI: 0.00, 0.69) as well as between age groups 10 years and older children (SMD for 12–14 years range 0.36–0.62).

The heterogeneity and global inconsistency were significantly high for EO (I2 = 79.8, 95% CI: 70.2, 86.3%, *χ*^2^ = 104.06, *df* = 21, *p* < 0.0001) and low for EC (I2 = 35.4, 95% CI: 0, 61.5%, *χ*^2^ = 32.53, *df* = 21, *p* = 0.0517). Inconsistency between designs appeared to be an issue in this subnetwork (*p* < 0.0001 for EO and EC). The results for detaching single designs show that the between-design heterogeneity can largely be traced back to the comparison of 4 vs. 5 for EO (*χ*2 = 46.45, *df* = 4, *p* < 0.0001) and EC (*χ*^2^ = 15.90, *df* = 4, *p* < 0.0001). Results for the full design-by-treatment interaction model show evidence of inconsistency between designs for EO (*χ*^2^ = 91.11, *df* = 5, *p* < 0.0001) and for EC (*χ*^2^ = 26.72, *df* = 5, *p* < 0.0001). Supplementary Tables S8C,D. summarizes the local inconsistency between direct and indirect evidence using the SIDE method for EO and EC. The results show 47 pairwise comparisons contributing both direct and indirect evidence. There is evidence of inconsistency for a few comparisons among age groups (in years) (*p* < 0.10 for 3 vs. 4, 3 vs. 5, 4 vs. 6, 5 vs. 8 for EO, and 5 vs. 7 for EC).

##### 3.3.2.3 Certainty of evidence

We estimated within-trial bias as the weighted average of the overall risk of bias and reporting bias was considered of some concern in the first subnetwork. Furthermore, we had no concerns about indirectness, and a clinically significant threshold was SMD = 0.20. For EO, no concern was reported about incoherence because no comparison had a global or local inconsistency. The certainty of the evidence was low or very low for the majority of the comparisons, mainly due to within-study bias and reporting bias (Supplementary Table S9A). For EC, some concerns were reported about incoherence because some comparisons had a global or local inconsistency. The certainty of the evidence was low or very low for the majority of the comparisons, mainly due to within-study bias and reporting bias (Supplementary Table S9B).

We estimated within-trial bias as the weighted average of the overall risk of bias, and reporting bias was considered low in the second subnetwork. Furthermore, we had no concerns about indirectness, and a clinically significant threshold was SMD = 1.0. We were concerned about incoherence because a few comparisons had a global or local inconsistency. The certainty of the evidence was low or very low for majority of the comparisons, mainly due to within-study bias and incoherence (Supplementary Table S9C). For EC, the certainty of the evidence was moderate or low for majority of the comparisons, mainly due to within-study bias and incoherence (Supplementary Table S9D).

##### 3.3.2.4 Publication bias

There was no clear evidence of publication bias according to a cluster robust Egger’s test for EO of sex comparisons (*p* = 0.46). For the funnel plot, see Supplementary Figure S3. We could not run this analysis for EC of sex comparisons after excluding the influential outliers (≥10 needed).

## 4 Discussion

### 4.1 Summary of main results

This review aimed to map age and gender-related reference values in a healthy population, assessed by computerized posturography in static and dynamic conditions, and discuss the strengths and limitations of these norms to outline future perspectives and needs. To the best of our knowledge, this work represents the first attempt to synthesize the literature on normative data for computerized posturography using a combined mixed method. The main findings of this systematic review are as follows: (1) Extensive heterogeneity was found in methodological characteristics, (2) there was insufficient risk of bias mitigation, (3) the majority of tasks evaluated less than four components of the SFPC, and (4) studies mostly used distance domain sway parameters and did not report the influence of other variables on postural sway.

Furthermore, we conducted quantitative analyses on the age- and sex-stratified data from these sway parameters of studies with similar interventions, comparisons, and groups. We included 17 studies for sex and age comparisons, involving 5,194 participants (females: 2,630; males: 2,564), in the multilevel or network meta-analyses. We could only conduct quantitative analysis for EO and EC conditions as there was an insufficient number of studies for other conditions. Based on the multilevel meta-analyses, females appeared to significantly outperform males in EC conditions. A significant difference was observed only in EC condition. Based on the network meta-analyses, we found a significant difference in sway among individuals between 50 and 79 years of age and younger individuals, with more instability observed in older participants in both EO and EC conditions. The results also revealed that younger children swayed significantly more than those aged between 8 and 14 years in EO condition. In EC condition, older children were significantly more stable than those aged 8 years and younger.

### 4.2 Methodological characteristics

We were able to draw some conclusions on methodological characteristics of normative data assessed by computerized posturography. Experimental standardization and reporting are lacking, leading to a low risk of bias mitigation. Specific details are provided in the following.

Only a few studies provided age-stratified reference values. However, some reported values per biological age, decade, or academic level, which made their comparison difficult. We also observed that various tasks and sway parameters were used, leading to semantic heterogeneity. The performances were most often evaluated through distance domain features. However, some questions have been raised about the relevance and reliability of these parameters (Hébert-Losier and Murray, 2020; Ruhe et al., 2010). Hébert-Losier and Murray (2020) found that the sway area and path of the center of pressure appeared to be the most reliable. However, an evaluation of the quality of the studies revealed that only four had good methodological quality. We also found that measurement conditions varied largely regarding arm and foot position, use of visual target, the number and duration of trials, and sequence of the conditions. The majority of studies did not report the influence of other variables (i.e., height, weight, foot placement) on postural sway. These factors need to be considered as they may influence postural control (i.e., anthropometric characteristics) (Chiari et al., 2002), feet positioning (Gibbons et al., 2019), and physical activity (Lelard and Ahmaidi, 2015). These discrepancies highlight methodological disparities that may impact the generalizability of these normative data. These findings agree with systematic reviews by Verbecque et al. (2016a,b) and Quijoux et al. (2020), where a need for standardization in posturography assessment was recommended for reliable and conclusive results.

Overall, participants in the included studies mostly represented regular individuals, with approximately equal numbers of females and males. The sample population largely consisted of children and young adults combined. Only a few studies investigated postural sway in elderly adults (Eriksen and Hougaard, 2023; Goble and Baweja, 2018b; Masui et al., 2005; Nishino et al., 2021; Perucca et al., 2021; Trueblood et al., 2018), as well as specific athletic (Goble et al., 2019b; Krityakiarana and Jongkamonwiwat, 2016; Massingale et al., 2018; Matsuda et al., 2010; Scaglioni-Solano and Aragón-Vargas, 2014; Schmidt et al., 2012; Weismiller et al., 2021) and military cohorts (Henry et al., 2022; Pletcher et al., 2017; Roberts et al., 2021). Age-related declines in balance have been widely reported in adults (Eriksen and Hougaard, 2023; Goble and Baweja, 2018b; Kollegger et al., 1992; Masui et al., 2005; Nishino et al., 2021; Perucca et al., 2021), yet few normative data are available on the geriatric population. This first observation demonstrated the under-representativeness of older adults, a critical gap given that this population is significantly more vulnerable to postural decline and related health issues. Older adults experience age-related changes in sensory, neuromuscular, and cognitive systems, which collectively impact balance and increase the risk of falls and injury. The research gap not only hinders the generalizability of findings to this high-risk group but also leaves clinicians without age-specific data that could inform tailored assessment, prevention, and treatment strategies. Additionally, this cohort did not include reference values for specific populations, such as athletes or military personnel, who may be at higher risk of developing balance impairments. Highly active individuals such as athletes and service members are at high risk of concussion, which can lead to functional impairments (Massingale et al., 2018). Highly active individuals, such as athletes and service persons, are at high risk of concussion that can lead to functional impairments (Massingale et al., 2018). As noted by Pletcher et al. (2017), differences in postural control can be observed across different military branches of the United States Special Operations Forces. The authors suggested that their training, mission environment, and equipment could have impacted their performances. Thus, reference values from civilian populations may not reflect the postural control of highly trained athletes or military personnel with higher and varying physical capacities. Age and sex-related reference values.

Sex-related instability has also been observed and appeared to be condition-dependent. We found that females were more stable than males in EC conditions. This has been corroborated in previous studies, which suggested that better control in females could be explained by physiologic and morphologic characteristics (Farenc et al., 2003; Kollegger et al., 1992). In EO condition, such differences are not observed due to the stabilizing effect of vision on postural sway in males.

Adults become more instable with age due to a natural decline in sensory systems’ functioning, which are less accurate and slower (Hageman et al., 1995). Eriksen and Hougaard (2023) demonstrated a progressive decline in SOT composite scores between healthy individuals aged over 70 and those aged between 20–39 years. Similarly, Perucca et al. (2021) and Cohen et al. (1996) demonstrated an influence of age on the scores of the same test. Their participants were divided into two older age groups: 80–84 and 85–89 years. The results revealed a significant effect of age on balance and sensory systems. These authors demonstrated that balance becomes less stable after the age of 85 years, and more marked changes were observed during vestibular-perturbed conditions. These age-related changes appeared more pronounced with sex as females were more stable (Goble and Baweja, 2018b; Kollegger et al., 1992; Masui et al., 2005). These results on the effect of age are corroborated by studies using other sway measures (Goble and Baweja, 2018b; Masui et al., 2005; Nishino et al., 2021).

### 4.3 Quality of the evidence: risk of bias and publication bias

The risk of bias in the included studies appears to be globally high. These results could be explained by the extensive variability in the methodological characteristics and lack of reporting. In fact, we found considerable evidence of heterogeneity in the sex-related comparisons for EO or in the age-related comparisons for EO in the second network involving children. We could only evaluate publication bias for EO conditions in sex-related comparisons as there was insufficient data for the other conditions and age-related comparisons. There was no clear evidence of publication bias. Overall, our confidence in the group estimates for age-related comparisons was low as we downgraded the certainty of the evidence for within-study bias and incoherence in comparisons involving children and for within-study bias and reporting bias in comparisons involving adults.

### 4.4 Strengths and limitations

We performed an extensive search, but some limitations must be considered. A potential limitation is the classification of some tasks and sway parameters based on definitions presented in the literature. In addition, some studies did not provide sufficient details to extract outcomes and adequately assess quality. Confidence in our results was only assessed using CINeMA (Nikolakopoulou et al., 2020), and care must be taken in interpreting these results as we included a small sample of studies in the quantitative analyses.

### 4.5 Implications for practice and research

This systematic review discussed the strengths and limitations of age and sex-related normative data and methodological characteristics to outline future perspectives and needs. We pointed out low methodological quality, sparse reporting, and variability in measurement characteristics. As noted, there was variability in the definition of tasks and sway parameters. The classification of these tasks showed that majority of the studies focused on static movement control (i.e., activity-based conditions), which is different from static measurement (external conditions). There is a need for reference values in dynamic movement control-based tasks (i.e., voluntary body movements). Furthermore, there could be a benefit in exploring the movements evaluated concerning the SFPC (Sibley et al., 2015). The MCT, the ADT, and the Concussion Balance Test (COBALT) evaluated the majority of the components of the SFPC (4 items). However, only one task was used in more than three studies, the MCT. The four SFPC components evaluated in the MCT were static stability, underlying motor systems, reactive postural control, and sensory integration. This framework can be used to identify underlying postural deficits for better patient management. Therefore, there is a need to evaluate balance using standardized tasks or designing future tasks encompassing the majority of the components of the SFPC to understand the underlying postural mechanisms. This recommendation also applies to exergaming interventions in the geriatric population (Tahmosybayat et al., 2018).

From a clinical perspective, there is a need for stratified normative data that considers normal aging processes separately for children, adults, and highly active individuals. Cohen et al. (1996) demonstrated that age-appropriate reference values per decade should be used for adults as postural control continues to decline up to the ninth decade. These deficits become more pronounced in high-risk concussion populations. It has been noted that differences could be expected among biological ages as children are still in the developing stages (Verbecque et al., 2016b). Therefore, reference values per decade for adults and biological ages for children might be necessary for better comparisons in clinical practice. Furthermore, our findings appeared to suggest a turning point in children and adults. Age-related differences point to improved stability for children approximately the age of 8 years, whereas adults swayed more from middle age and upward.

## 5 Conclusion

In summary, our results revealed sex and age-related effects on balance, with females demonstrating more stability than males in EC conditions. In addition, we also observed that children younger than 8 years of age and adults over 50 years of age swayed more in EO condition and EC, suggesting a turning point. To improve future research and clinical applicability, we recommend that studies stratify reference values by age and sex and include detailed participant information (e.g., anthropometric data) to allow for meaningful comparisons. Standardizing experimental conditions and harmonizing sway parameters will also enhance the reliability and comparability of findings across studies. Finally, evidence-based postural impairment management requires age- and sex-related normative data to inform targeted interventions and improve outcomes.

## Data Availability

The raw data supporting the conclusions of this article will be made available by the authors, without undue reservation.

## Footnotes

^1^ https://www.r-project.org/

## References

[ref1] Achour Lebib S. Ben B. Missaoui I. Miri F. Z. Salah B. Dziri C. (2006). Rôle Du Neurocom Balance Master^®^ Dans l’évaluation Des Troubles de l’équilibre et Du Risque de Chute Chez Le Sujet Âgé. Annal. Readapt. Med. Physiq. 49, 210–217. doi: 10.1016/j.annrmp.2006.03.005, PMID: 16675055

[ref2] Albertsen I. M. Ghédira M. Gracies J. M. Hutin É. (2017). Postural stability in young healthy subjects – impact of Reduced Base of support, visual deprivation, dual tasking. J. Electromyogr. Kinesiol. 33, 27–33. doi: 10.1016/j.jelekin.2017.01.005 28135586

[ref3] Alburquerque-Sendín F. Fernández-de-las-Peñas C. Santos-del-Rey M. Martín-Vallejo F. J. (2009). Immediate effects of bilateral manipulation of Talocrural joints on standing stability in healthy subjects. Man. Ther. 14, 75–80. doi: 10.1016/j.math.2007.11.005, PMID: 18280767

[ref4] Alonso A. C. Natália Mariana S. Luna L. M. Barbieri F. Santos S. Greve J. M. D. (2012). The influence of anthropometric factors on postural balance: the relationship between body composition and Posturographic measurements in young adults. Clinics 67, 1433–1441. doi: 10.6061/clinics/2012(12)14, PMID: 23295598 PMC3521807

[ref5] Balduzzi S. Rücker G. Nikolakopoulou A. Papakonstantinou T. Salanti G. Efthimiou O. . (2023). Netmeta: an R package for network Meta-analysis using frequentist methods. J. Stat. Softw. 106, 1–40. doi: 10.18637/jss.v106.i0237138589

[ref6] Baloh R. W. Jacobson K. M. Beykirch K. Honrubia V. (1998). Static and dynamic Posturography in patients with vestibular and cerebellar lesions. Arch. Neurol. 55, 649–654. doi: 10.1001/archneur.55.5.649, PMID: 9605721

[ref7] Barozzi S. Socci M. Soi D. Di Berardino F. Fabio G. Forti S. . (2014). Reliability of postural control measures in children and young adolescents. Eur. Arch. Otorrinolaringol. 271, 2069–2077. doi: 10.1007/s00405-014-2930-9, PMID: 24557440

[ref8] Beauchet O. Barden J. Liu-Ambrose T. Chester V. L. Szturm T. Allali G. (2016). The relationship between hippocampal volume and static postural sway: results from the GAIT study. Age 38, 19–18. doi: 10.1007/s11357-016-9883-4, PMID: 26833034 PMC5005866

[ref9] Berg K. O. Maki B. E. Williams J. I. Holliday P. J. Wood-Dauphinee S. L. (1992). Clinical and laboratory measures of postural balance in an elderly population. Arch. Phys. Med. Rehabil. 73, 1073–1080, PMID: 1444775

[ref10] Bizzo G. Guillet N. Patat A. Gagey P. M. (1985). Specifications for building a vertical force platform designed for clinical Stabilometry. Med. Biol. Eng. Comput. 23, 474–476. doi: 10.1007/BF02448937, PMID: 4068783

[ref11] Black F. O. Shupert C. L. Horak F. B. Nashner L. M. (1988). Abnormal postural control associated with peripheral vestibular disorders. Prog. Brain Res. 76, 263–275. doi: 10.1016/S0079-6123(08)64513-6 3265212

[ref12] Black F. O. Wall C. Nashner L. M. (1983). Effects of visual and support surface orientation references upon postural control in vestibular deficient subjects. Acta Otolaryngol. 95, 199–210. doi: 10.3109/00016488309130936 6601353

[ref13] Black F. O. Wall C. Rockette H. E. Kitch R. (1982). Normal subject postural sway during the Romberg test. Am. J. Otolaryngol. 3, 309–318. doi: 10.1016/S0196-0709(82)80002-1, PMID: 7149143

[ref14] Bloem B. R. Visser J. E. Allum J. H. J. (2003). “Chapter 20 Posturography” in Handbook of clinical neurophysiology. (Ed.) Mark, H. (Elsevier), 295–336.

[ref15] Carrick F. R. Oggero E. Pagnacco G. (2007). Posturographic changes associated with music listening. J. Altern. Complement. Med. 13, 519–526. doi: 10.1089/acm.2007.7020, PMID: 17604555

[ref16] Casselbrant M. L. Mandel E. M. Sparto P. J. Perera S. Redfern M. S. Fall P. A. . (2010). Longitudinal Posturography and rotational testing in children three to nine years of age: normative data. Otolaryngology 142, 708–714. doi: 10.1016/j.otohns.2010.01.028, PMID: 20416461 PMC2900920

[ref17] Charpiot A. Tringali S. Ionescu E. Vital-Durand F. Ferber-Viart C. (2010). Vestibulo-ocular reflex and balance maturation in healthy children aged from six to twelve years. Audiol. Neurotol. 15, 203–210. doi: 10.1159/000255338 19893301

[ref18] Chiari L. Rocchi L. Cappello A. (2002). Stabilometric parameters are affected by anthropometry and foot placement. Clin. Biomech. 17, 666–677. doi: 10.1016/S0268-0033(02)00107-9, PMID: 12446163

[ref19] Clark R. A. Mentiplay B. F. Pua Y. H. Bower K. J. (2018). Reliability and validity of the Wii balance Board for Assessment of standing balance: a systematic review. Gait Posture 61, 40–54. doi: 10.1016/j.gaitpost.2017.12.022 29304510

[ref20] Cohen H. Heaton L. G. Congdon S. L. Jenkins H. A. (1996). Changes in sensory organization test scores with age. Age Ageing 25, 39–44. doi: 10.1093/ageing/25.1.39 8670527

[ref21] de la Torre J. Marin J. Marin J. J. Auria J. M. Sanchez-Valverde M. B. (2017). Balance study in asymptomatic subjects: determination of significant variables and reference patterns to improve clinical application. J. Biomech. 65, 161–168. doi: 10.1016/j.jbiomech.2017.10.013, PMID: 29126606

[ref22] Di Fabio R. P. (1995). Sensitivity and specificity of platform Posturography for identifying patients with vestibular dysfunction. Phys. Ther. 75, 290–305. doi: 10.1093/ptj/75.4.290 7899487

[ref23] Di Fabio R. P. (1996). Meta-analysis of the sensitivity and specificity of platform Posturography. Arch. Otolaryngol. Head Neck Surg. 122, 150–156. doi: 10.1001/archotol.1996.01890140036008, PMID: 8630208

[ref24] di Fabio R. P. Foudriat B. A. (1996). Responsiveness and reliability of a pediatric strategy score for balance. Physiother. Res. Int. 1, 180–194. doi: 10.1002/pri.57, PMID: 9238733

[ref25] Domènech-Vadillo E. Aguilera-Aguilera G. Sánchez-Blanco C. Batuecas-Caletrio Á. Guajardo C. Pérez N. . (2019). Normative data for static balance testing in healthy individuals using open source computerized Posturography. Eur. Arch. Otorrinolaringol. 276, 41–48. doi: 10.1007/s00405-018-5170-6, PMID: 30327905

[ref26] Eriksen N. D. Hougaard D. D. (2023). Age- and gender-specific normative data on computerized dynamic Posturography in a cohort of Danish adults. Eur. Arch. Otorrinolaringol. 280, 2191–2200. doi: 10.1007/s00405-022-07706-y, PMID: 36326952

[ref27] Faraldo-García A. Santos-Pérez S. Crujeiras R. Soto-Varela A. (2016). Postural changes associated with ageing on the sensory organization test and the limits of stability in healthy subjects. Auris Nasus Larynx 43, 149–154. doi: 10.1016/j.anl.2015.07.001, PMID: 26254957

[ref28] Faraldo-García A. Santos-Pérez S. Crujeiras-Casais R. Labella-Caballero T. Soto-Varela A. (2012). Influence of age and gender in the sensory analysis of balance control. Eur. Arch. Otorrinolaringol. 269, 673–677. doi: 10.1007/s00405-011-1707-7, PMID: 21789678

[ref29] Farenc I. Rougier P. Berger L. (2003). The influence of gender and body characteristics on upright stance. Ann. Hum. Biol. 30, 279–294. doi: 10.1080/0301446031000068842, PMID: 12850961

[ref30] Fujimoto C. Murofushi T. Chihara Y. Ushio M. Sugasawa K. Yamaguchi T. . (2009). Assessment of diagnostic accuracy of foam Posturography for peripheral vestibular disorders: analysis of parameters related to visual and somatosensory dependence. Clin. Neurophysiol. 120, 1408–1414. doi: 10.1016/j.clinph.2009.05.002, PMID: 19520601

[ref31] García-Soidán J. L. García-Liñeira J. Leirós-Rodríguez R. Soto-Rodríguez A. (2020). Physical activity practice and optimal development of postural control in school children: are they related? J. Clin. Med. 9:2919. doi: 10.3390/jcm9092919, PMID: 32927763 PMC7565156

[ref32] Gauchard G. (2003). Physical activity improves gaze and posture control in the elderly. Neurosci. Res. 45, 409–417. doi: 10.1016/S0168-0102(03)00008-7 12657454

[ref33] Gibbons C. T. Amazeen P. G. Likens A. D. (2019). Effects of foot placement on postural sway in the anteroposterior and Mediolateral directions. Mot. Control. 23, 149–170. doi: 10.1123/mc.2017-0074, PMID: 30518285

[ref34] Goble D. J. Baweja H. S. (2018a). Normative data for the BTrackS balance test of postural sway: Results from 16,357 community-dwelling individuals who were 5 to 100 years old. Phys. Ther. 98, 779–785. doi: 10.1093/ptj/pzy062 29788179

[ref35] Goble D. J. Baweja H. S. (2018b). Postural sway normative data across the adult lifespan: results from 6280 individuals on the balance tracking system balance test. Geriatr. Gerontol. Int. 18, 1225–1229. doi: 10.1111/ggi.13452, PMID: 29897159

[ref36] Goble D. J. Brar H. Brown E. C. Marks C. R. Baweja H. S. (2019a). Normative data for the balance tracking system modified clinical test of sensory integration and balance protocol. Med. Dev. 12, 183–191. doi: 10.2147/MDER.S206530, PMID: 31191047 PMC6519013

[ref37] Goble D. J. Rauh M. J. Baweja H. S. (2019b). Normative data for the Btracks balance test concussion-management tool: results from 10045 athletes aged 8 to 21 years. J. Athl. Train. 54, 439–444. doi: 10.4085/1062-6050-178-18, PMID: 30870601 PMC6522082

[ref38] Hageman P. A. Michael Leibowitz J. Blanke D. (1995). Age and gender effects on postural control measures. Arch. Phys. Med. Rehabil. 76, 961–965. doi: 10.1016/S0003-9993(95)80075-1 7487439

[ref39] Hamid M. A. Hughes G. B. Kinney S. E. (1991). Specificity and sensitivity of dynamic Posturography: a retrospective analysis. Acta Otolaryngol. 111, 596–600. doi: 10.3109/00016489109131480 1927480

[ref40] Hébert-Losier K. Murray L. (2020). Reliability of Centre of Pressure, plantar pressure, and plantar-flexion isometric strength measures: a systematic review. Gait Posture 75, 46–62. doi: 10.1016/j.gaitpost.2019.09.027, PMID: 31593873

[ref41] Henry N. E. Weart A. N. Miller E. M. Feltner L. D. Goss D. L. (2022). Normative data for the NeuroCom sensory organization test in United States military academy boxers. Int. J. Sports Phys. Ther. 17, 366–377. doi: 10.26603/001c.32547 35391868 PMC8975572

[ref42] Herssens N. Verbecque E. McCrum C. Meijer K. van de Berg R. Saeys W. . (2020). A systematic review on balance performance in patients with bilateral Vestibulopathy. Phys. Ther. 100, 1582–1594. doi: 10.1093/ptj/pzaa083, PMID: 32367131

[ref43] Higgins J. P. T. Green S. Van Den A. B. (2020). Cochrane handbook for systematic reviews of interventions. Int. Coach. Psychol. Rev. 15:123. doi: 10.53841/bpsicpr.2020.15.2.123

[ref44] Horak F. B. Nashner L. M. Diener H. C. (1990). Postural strategies associated with somatosensory and vestibular loss. Exp. Brain Res. 82, 167–177. doi: 10.1007/BF00230848, PMID: 2257901

[ref45] Ionescu E. Dubreuil C. Ferber-Viart C C. (2005). Physiological changes in balance control of adults aged 20 to 60 years assessed with Equitest. Ann. Otolaryngol. 122, 231–235. doi: 10.1016/S0003-438X(05)82354-0 16439933

[ref46] Kollegger H. Baumgartner C. Wöber C. Oder W. Deecke L. (1992). Spontaneous body sway as a function of sex, age, and vision: Posturographic study in 30 healthy adults. Eur. Neurol. 32, 253–259. doi: 10.1159/000116836, PMID: 1521545

[ref47] Kollegger H. Wöber C. Baumgartner C. Deecke L. (1989). Stabilizing and destabilizing effects of vision and foot position on body sway of healthy young subjects: a Posturographic study. Eur. Neurol. 29, 241–245. doi: 10.1159/000116420 2792141

[ref48] Krityakiarana W. Jongkamonwiwat N. (2016). Comparison of balance performance between Thai classical dancers and non-dancers. J. Dance Med. Sci. 20, 72–78. doi: 10.12678/1089-313X.20.2.72, PMID: 27245946

[ref49] Lara S. Graup S. Balk R. S. Teixeira L. P. Farias A. D. Alves G. B. . (2018). Association between postural balance and anthropometric indexes in elementary schoolchildren. Rev. Paul. Pediatr. 36, 7–65. doi: 10.1590/1984-0462/;2018;36;1;00011, PMID: 29160409 PMC5849375

[ref50] Lelard T. Ahmaidi S. (2015). Effects of physical training on age-related balance and postural control. Neurophysiol. Cliniq. 45, 357–369. doi: 10.1016/J.NEUCLI.2015.09.008 26548366

[ref51] Letz R. Fredric Gerr F. Harris-abbott D. Robert Dick F. Gerr D. H.-a. Dick R. (1996). A comparison of standing steadiness measurements from two devices: covariates and Normal values. Neurotoxicol. Teratol. 18, 83–88. doi: 10.1016/0892-0362(95)02012-8, PMID: 8700047

[ref52] Libardoni T. de Cássia C. da Silveira B. Sinhorim L. M. B. Siriani A. de Oliveira M. . (2018). Reference values and equations reference of balance for children of 8 to 12 years. Gait Posture 60, 122–127. doi: 10.1016/j.gaitpost.2017.11.004 29190542

[ref53] Macedo C. Gazzola J. M. Ricci N. A. Doná F. Ganança F. F. (2015). Influence of sensory information on static balance in older patients with vestibular disorder. Braz. J. Otorhinolaryngol. 81, 50–57. doi: 10.1016/j.bjorl.2014.11.004, PMID: 25554561 PMC9452215

[ref54] Massingale S. L. Alexander A. D. Erickson S. M. McQueary E. S. Gerkin R. D. Schodrof S. B. . (2018). Assessing balance in an athletic population: normative data for the concussion balance test (COBALT©). Int. J. Athletic Therapy Train. 23, 96–100. doi: 10.1123/ijatt.2017-0042

[ref55] Masui T. Hasegawa Y. Matsuyama Y. Sakano S. Kawasaki M. Suzuki S. (2005). Gender differences in platform measures of balance in rural community-dwelling elders. Arch. Gerontol. Geriatr. 41, 201–209. doi: 10.1016/j.archger.2005.02.003, PMID: 16085072

[ref56] Matsuda S. Demura S. Demura T. (2010). Examining differences between Center of Pressure Sway in one-legged and two-legged stances for soccer players and typical adults. Percept. Mot. Skills 110, 751–760. doi: 10.2466/pms.110.3.751-760, PMID: 20681329

[ref57] Micarelli A. Viziano A. Augimeri I. Micarelli B. Alessandrini M. (2020). Age-related assessment of postural control development: a cross-sectional study in children and adolescents. J. Mot. Behav. 52, 418–426. doi: 10.1080/00222895.2019.1643284, PMID: 31328659

[ref58] Mnejja K. Fendri T. Chaari F. Harrabi M. A. Sahli S. (2022). Reference values of postural balance in preschoolers: age and gender differences for 4–5 years old Tunisian children. Gait Posture 92, 401–406. doi: 10.1016/j.gaitpost.2021.12.015, PMID: 34959208

[ref59] Nashner L. M. (1982). Adaptation of human movement to altered environments. Trends Neurosci. 5, 358–361. doi: 10.1016/0166-2236(82)90204-1

[ref60] Nashner L. Owen Black F. Lilly D. J. (1994). Apparatus and method for determining the presence of vestibular pathology. Available at: https://patents.google.com/patent/WO1988004909A2/en (Accessed May 1, 2023).

[ref61] Nashner L. M. Peters J. F. (1990). Dynamic Posturography in the diagnosis and Management of Dizziness and Balance Disorders. Neurol. Clin. 8, 331–349. doi: 10.1016/S0733-8619(18)30359-1 2193215

[ref9001] Nikolakopoulou A. Higgins J. P. T. Papakonstantinou T. Chaimani A. Del Giovane C. Egger M. (2020). CINeMA: An approach for assessing confidence in the results of a network meta-analysis. PLoS Medicine 17, 1–19. 10.1371/journal.pmed.1003082 PMC7122720 32243458

[ref62] Nishino L. K. Rocha G. D. Almeida T. S. de Souza F. Ribeiro A. Q. Cóser P. L. (2021). Protocol for static Posturography with dynamic tests in individuals without vestibular complaints using the Horus system. CODAS 33, 1–13. doi: 10.1590/2317-1782/20202019270, PMID: 34161438

[ref63] Ouzzani M. Hammady H. Fedorowicz Z. Elmagarmid A. (2016). Rayyan-a web and Mobile app for systematic reviews. Syst. Rev. 5:384. doi: 10.1186/s13643-016-0384-4, PMID: 27919275 PMC5139140

[ref64] Owen N. Leadbetter A. G. Yardley L. (1998). Relationship between postural control and motion sickness in healthy subjects. Brain Res. Bull. 47, 471–474. doi: 10.1016/S0361-9230(98)00101-4, PMID: 10052576

[ref65] Paillard T. Noé F. (2015). Techniques and methods for testing the postural function in healthy and pathological subjects. Biomed. Res. Int. 2015, 1–15. doi: 10.1155/2015/891390, PMID: 26640800 PMC4659957

[ref66] Patti A. Bianco A. Şahin N. Sekulic D. Paoli A. Iovane A. . (2018). Postural control and balance in a cohort of healthy people living in Europe an observational study. Medicine 97:e13835. doi: 10.1097/MD.0000000000013835, PMID: 30593180 PMC6314740

[ref67] Perucca L. Majnardi A. R. Frau S. Scarano S. (2021). Normative data for the NeuroCom® sensory organization test in subjects aged 80–89 years. Front. Hum. Neurosci. 15:761262. doi: 10.3389/fnhum.2021.761262, PMID: 34867246 PMC8641293

[ref68] Pletcher E. R. Williams V. Abt J. P. Morgan P. M. Parr J. J. Wohleber M. F. . (2017). Normative data for the Neurocom sensory organization test in us military special operations forces. J. Athl. Train. 52, 129–136. doi: 10.4085/1062-6050-52.1.05, PMID: 28140624 PMC5343525

[ref69] Podsiadlo J. D. Bscpt S. Richardson M. D. J. (1991). The timed ‘up & go’: a test of basic functional Mobilitv for frail Elderlv persons. J. Am. Geriatr. Soc. 39, 142–148. doi: 10.1111/j.1532-5415.1991.tb01616.x 1991946

[ref70] Pollock A. S. Durward B. R. Rowe P. J. Paul J. P. (2000). What is balance? Clin. Rehabil. 14, 402–406. doi: 10.1191/0269215500cr342oa 10945424

[ref71] Prieto T. E. Myklebust J. B. Hoffmann R. G. Lovett E. G. Myklebust B. M. (1996). Measures of postural steadiness: differences between healthy young and elderly adults. IEEE Trans. Biomed. Eng. 43, 956–966. doi: 10.1109/10.532130, PMID: 9214811

[ref9002] Pustejovsky J. (2024). ClubSandwich: Cluster-Robust (Sandwich) Variance Estimators with Small-Sample Corrections. R package version 0.5.11.9999. Available at: http://jepusto.github.io/clubSandwich

[ref72] Quijoux F. Nicolaï A. Chairi I. Bargiotas I. Ricard D. Yelnik A. . (2021). A review of Center of Pressure (COP) variables to quantify standing balance in elderly people: algorithms and open-access code. Physiol. Rep. 9:15067. doi: 10.14814/phy2.15067, PMID: 34826208 PMC8623280

[ref73] Quijoux F. Vienne-Jumeau A. Bertin-Hugault F. Zawieja P. Lefevre M. Vidal P. P. . (2020). Center of Pressure Displacement Characteristics Differentiate Fall Risk in older people: a systematic review with Meta-analysis. Ageing. Res. Rev. 62:101117. doi: 10.1016/j.arr.2020.101117 32565327

[ref74] Roberts H. J. Hoppes C. W. Del Toro Y. M. Lambert K. H. Springer B. A. (2021). Normative values for the sensory organization test in an active duty military cohort. Gait Posture 85, 31–37. doi: 10.1016/j.gaitpost.2021.01.014, PMID: 33513530

[ref75] Ruhe A. Fejer R. Walker B. (2010). The test-retest reliability of Centre of Pressure Measures in bipedal static task conditions - a systematic review of the literature. Gait Posture 32, 436–445. doi: 10.1016/j.gaitpost.2010.09.012, PMID: 20947353

[ref76] Sackley C. M. Lincoln N. B. (1991). Weight distribution and postural sway in healthy adults. Clin. Rehabil. 5, 181–186. doi: 10.1177/026921559100500302

[ref77] Scaglioni-Solano P. Aragón-Vargas L. F. (2014). Validity and reliability of the Nintendo Wii balance board to assess standing balance and sensory integration in highly functional older adults. Int. J. Rehabil. Res. 37, 138–143. doi: 10.1097/MRR.0000000000000046 24445863

[ref78] Schmidt J. D. Register-Mihalik J. K. Mihalik J. P. Kerr Z. Y. Guskiewicz K. M. (2012). Identifying impairments after concussion: normative data versus individualized baselines. Med. Sci. Sports Exerc. 44, 1621–1628. doi: 10.1249/MSS.0b013e318258a9fb 22525765

[ref79] Shams A. Vameghi R. Dehkordi P. S. Allafan N. Bayati M. (2020). The development of postural control among children: repeatability and normative data for computerized dynamic Posturography system. Gait Posture 78, 40–47. doi: 10.1016/j.gaitpost.2020.03.002, PMID: 32200162

[ref80] Sibley K. M. Beauchamp M. K. Van Ooteghem K. Straus S. E. Jaglal S. B. (2015). Using the systems framework for postural control to analyze the components of balance evaluated in standardized balance measures: a scoping review. Arch. Phys. Med. Rehabil. 96:e29, 122–132. doi: 10.1016/j.apmr.2014.06.021 25073007

[ref81] Sinno S. Dumas G. Mallinson A. Najem F. Abouchacra K. S. Nashner L. . (2021). Changes in the sensory weighting strategies in balance control throughout maturation in children. J. Am. Acad. Audiol. 32, 122–136. doi: 10.1055/s-0040-1718706, PMID: 33296934

[ref82] Tahmosybayat R. Baker K. Godfrey A. Caplan N. Barry G. (2018). Movements of older adults during exergaming interventions that are associated with the systems framework for postural control: a systematic review. Maturitas 111, 90–99. doi: 10.1016/j.maturitas.2018.03.005, PMID: 29673837

[ref83] Tinetti M. E. (1986). Performance-oriented assessment of mobility problems in elderly patients. J. Am. Geriatr. Soc. 34, 119–126. doi: 10.1111/j.1532-5415.1986.tb05480.x, PMID: 3944402

[ref84] Trueblood P. R. Rivera M. Lopez C. Bentley C. Wubenhorst N. (2018). Age-based normative data for a computerized dynamic Posturography system that uses a virtual visual surround environment. Acta Otolaryngol. 138, 597–602. doi: 10.1080/00016489.2018.1429653 29390922

[ref85] Verbecque E. Lobo P. H. da Costa P. Meyns K. D. Vereeck L. Hallemans A. (2016a). Age-related changes in postural sway in preschoolers. Gait Posture 44, 116–122. doi: 10.1016/j.gaitpost.2015.11.016, PMID: 27004643

[ref86] Verbecque E. Vereeck L. Hallemans A. (2016b). Postural sway in children: a literature review. Gait Posture 49, 402–410. doi: 10.1016/j.gaitpost.2016.08.003, PMID: 27505144

[ref87] Viechtbauer W. (2010). Conducting Meta-analyses inRwith themetaforPackage. JSS 36, 1–48. doi: 10.18637/jss.v036.i03

[ref88] Wall C. Owen Black F. Wall C. (1983). Postural stability and rotational tests: their effectiveness for screening dizzy patients. Acta Otolaryngol. 95, 235–246. doi: 10.3109/00016488309130940, PMID: 6601356

[ref89] Weismiller S. A. Monaco R. Womack J. Alderman B. Esopenko C. Conway F. N. . (2021). Individual baseline balance assessments in a large sample of incoming NCAA division I athletes using a force plate system. Int. J. Sports Phys. Ther. 16, 126–133. doi: 10.26603/001c.18713, PMID: 33604142 PMC7872460

[ref90] Whiting P. Rutjes A. W. Reitsma J. B. Bossuyt P. M. Kleijnen J. (2003) The development of QUADAS: a tool for the quality assessment of studies of diagnostic accuracy included in systematic reviews. Available at: http://www.biomedcentral.com/1471-2288/3/25 10.1186/1471-2288-3-25 PMC305345 14606960

[ref91] Whiting P. F. Weswood M. E. Rutjes A. W. S. Reitsma J. B. Bossuyt P. N. M. Kleijnen J. (2006). Evaluation of QUADAS, a tool for the quality assessment of diagnostic accuracy studies. BMC Med. Res. Methodol. 10:25. doi: 10.1186/1471-2288-3-25, PMID: 16519814 PMC1421422

