# Supplementary material for: Normative data for instrumented posturography: a systematic review and meta-analysis
Source: Front Hum Neurosci. 2024 Dec 18;18:1498107. doi: 10.3389/fnhum.2024.1498107 (PMC11688309; doi:10.3389/fnhum.2024.1498107)
Supplement: Supplementary file 1 [file Data_Sheet_1.DOCX]

**SUPPLEMENTARY INFORMATION**

| **SECTIONS** | **PAGES** |
| --- | --- |
| **Section A. Supplementary details on participant characteristics.** |  |
| Table S1. Methodological characteristics of studies included in systematic review. | 2-6 |
| Table S2. Quality assessment, using the QUADAS, of studies included in systematic review. | 7-8 |
| Figure S2. Frequency of studies reporting other variables. | 9 |
| **Section B. Multilevel meta-analysis using robust variance estimation (RVE) results.** |  |
| Table S4. Sensitivity of meta-analysis parameters estimates in EO and EC. | 10 |
| Figure S3. Funnel plot in EO condition. | 11 |
| Table S5. Subgroup analyses for EO condition. | 11 |
| **Section C. Frequentist network meta-analysis.** |  |
| Table S6. Distribution of potential effect modifiers in EO and EC conditions. | 12-15 |
| Figure S4. Subnetworks for different age comparisons A) in EO and B) EC conditions. | 16 |
| Table S7. Direct and indirect evidence and network meta-analysis summary table of the first subnetwork for both conditions. | 16 |
| Table S8. Direct and indirect evidence and network meta-analysis summary table of the second subnetwork for both conditions. | 17-19 |
| Table S9. The analysis of Separate indirect from direct evidence (SIDE) (back-calculation method). | 19-22 |
| Table S10. CINeMA grading of the certainty of the evidence. | 23-32 |

**Section A. Supplementary details on participant characteristics**

**Table S1. Methodological characteristics of studies included in systematic review.**

**Table S2. Quality assessment, using the QUADAS, of studies included in systematic review.**

| **References** | **Q1** | **Q2** | **Q5** | **Q9** | **Q12** | **Q13** | **Total** | **Quality** |
| --- | --- | --- | --- | --- | --- | --- | --- | --- |
| Albertsen et al., 2017 | N | Y | N | Y | Y | N | 3 | low |
| Alburquerque et al., 2009 | N | Y | N | N | Y | unclear | 2 | low |
| Barozzi et al., 2014 | N | Y | N | Y | Y | unclear | 3 | low |
| Beauchet et al., 2016 | N | Y | N | Y | Y | unclear | 3 | low |
| Black et al., 1982 | N | Y | N | unclear | Y | unclear | 2 | low |
| Carrick et al., 2007 | N | N | N | N | Y | N | 1 | low |
| Casselbrant et al., 2010 | N | Y | N | unclear | Y | Y | 3 | low |
| Charpiot et al., 2009 | N | Y | N | N | Y | N | 2 | low |
| de la Torre et al., 2017 | N | Y | N | Y | Y | N | 3 | low |
| DiFabio et al., 1996 | N | Y | N | Y | Y | N | 3 | low |
| Domènech-Vadillo et al., 2019 | unclear | Y | N | Y | Y | Y | 4 | high |
| Eriksen et al, 2023 | N | Y | N | Y | Y | unclear | 3 | low |
| Faraldo-Garcia et al., 2012 | N | Y | Y | N | Y | N | 3 | low |
| Faraldo-Garcia et al., 2016 | N | Y | Y | N | Y | N | 3 | low |
| Goble & Baweja, 2018 | Y | N | N | Y | Y | unclear | 3 | low |
| Goble & Baweja, 2018 | Y | N | N | Y | Y | unclear | 3 | low |
| Goble et al., 2019 | N | N | N | Y | Y | Y | 3 | low |
| Goble et al., 2019 | Y | N | N | Y | Y | unclear | 3 | low |
| Henry et al., 2022 | N | N | N | Y | Y | unclear | 2 | low |
| Ionescu et al., 2006 | N | Y | N | Y | Y | Y | 4 | high |
| Kolleger et al., 1992 | N | Y | unclear | Y | Y | unclear | 3 | low |
| Krityakiarana et al., 2016 | N | Y | N | Y | Y | unclear | 3 | low |
| Lara et al., 2018 | N | Y | N | Y | Y | unclear | 3 | low |
| Letz et al., 1996 | N | Y | N | Y | Y | N | 3 | low |
| Libardoni et al., 2018 | N | Y | N | Y | Y | N | 3 | low |
| Massingale et al., 2018 | N | N | unclear | unclear | Y | Y | 2 | low |
| Masui et al., 2005 | Y | Y | Y | Y | Y | Y | 6 | high |
| Matsuda et al., 2010 | N | N | N | Y | Y | unclear | 2 | low |
| Micarelli et al., 2020 | N | Y | Y | unclear | Y | unclear | 3 | low |
| Mnejja et al., 2022 | Y | Y | Y | Y | Y | unclear | 5 | high |
| Nishino et al., 2021 | Y | Y | N | Y | Y | Y | 5 | high |
| Owen et al., 2008 | N | N | N | Y | Y | unclear | 2 | low |
| Patti et al., 2018 | Y | Y | N | Y | Y | Y | 5 | high |
| Perucca et al., 2021 | N | Y | Y | Y | Y | unclear | 4 | high |
| Pletcher et al., 2017 | Y | N | N | unclear | Y | Y | 3 | low |
| Roberts et al., 2021 | N | Y | N | Y | Y | N | 3 | low |
| Sackley & Lincoln, 1991 | N | Y | N | unclear | Y | N | 2 | low |
| Scaglioni et al., 2014 | N | Y | N | unclear | N | unclear | 1 | low |
| Schmidt et al., 2012 | N | Y | N | Y | Y | Y | 4 | high |
| Shams et al., 2020 | Y | Y | N | Y | Y | Y | 5 | high |
| Sinno et al., 2021 | N | Y | Y | unclear | Y | unclear | 3 | low |
| Trueblood et al., 2018 | N | Y | N | Y | Y | unclear | 3 | low |
| Verbecque et al., 2016 | N | Y | N | Y | Y | Y | 4 | high |
| Weismiller et al., 2021 | N | Y | N | unclear | Y | unclear | 2 | low |

**A**

**B**

**Figure S2. Frequency of studies reporting other variables for studies included in A) the systematic review and B) excluded from meta-analyses.** The other variables reported are plotted according to the number of studies and grouped by population (children, adults, or both). The effects of each factor were categorized as “NS” for non-significant or “S” for significant and not reported information was scored as “NP”. A decrease represented less sway. When the effets were non applicable, it was noted as ‘NA’.


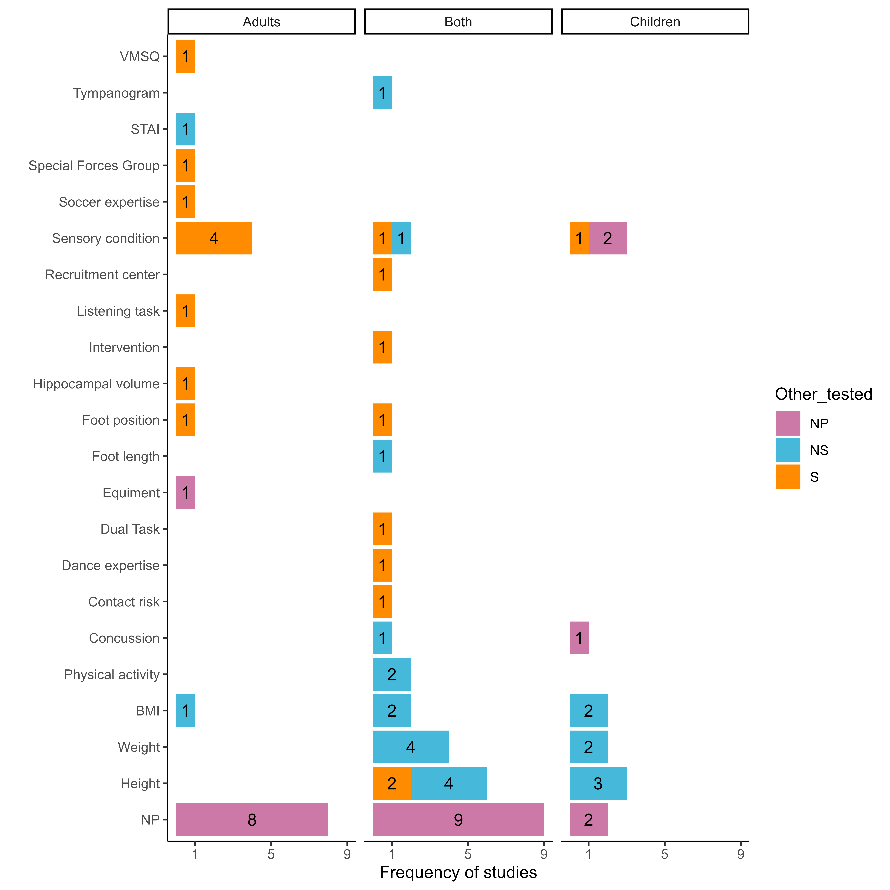

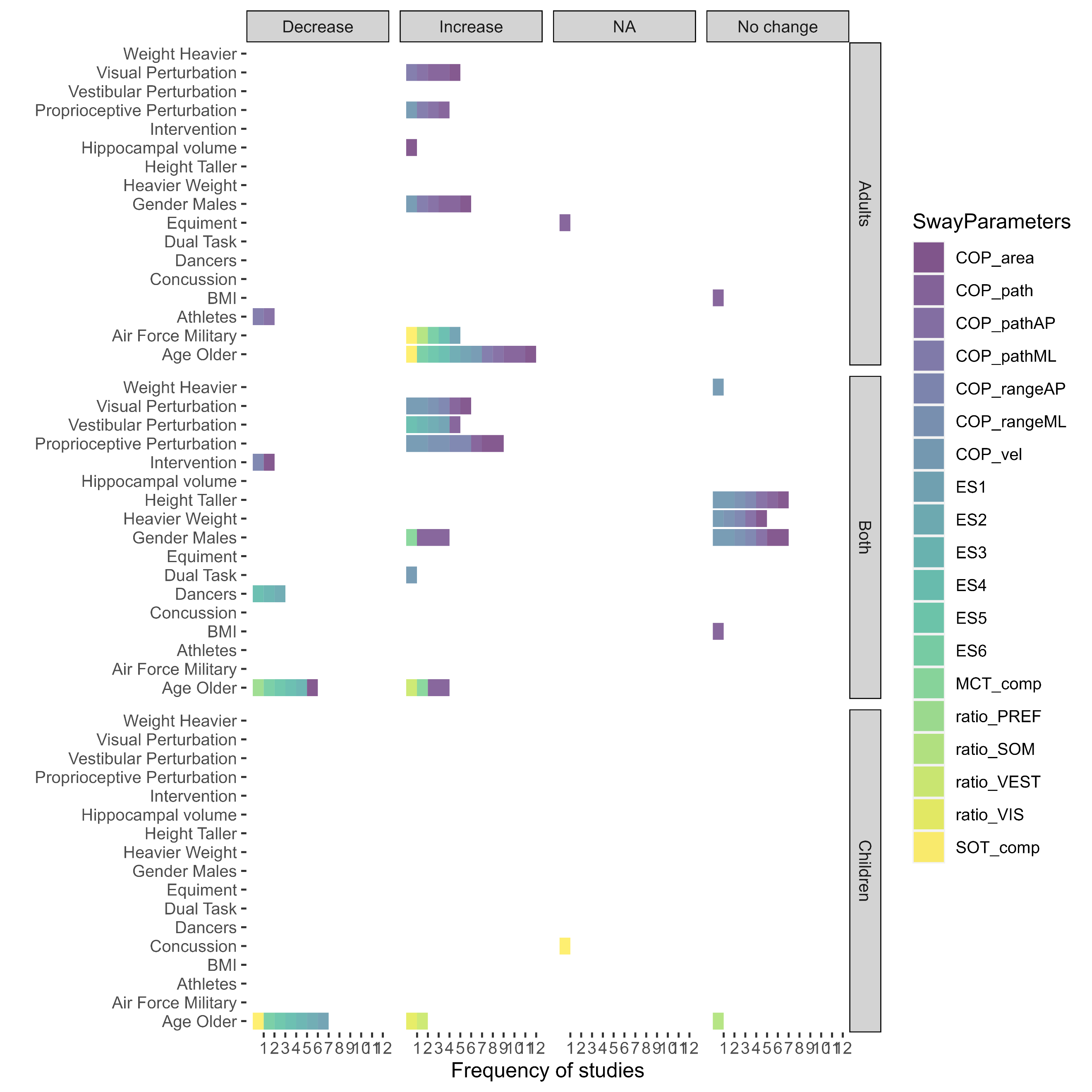


**Section B. Multilevel meta-analysis using robust variance estimation (RVE) results.**

**Table S4. Sensitivity of meta-analysis parameters estimates in EO and EC.**


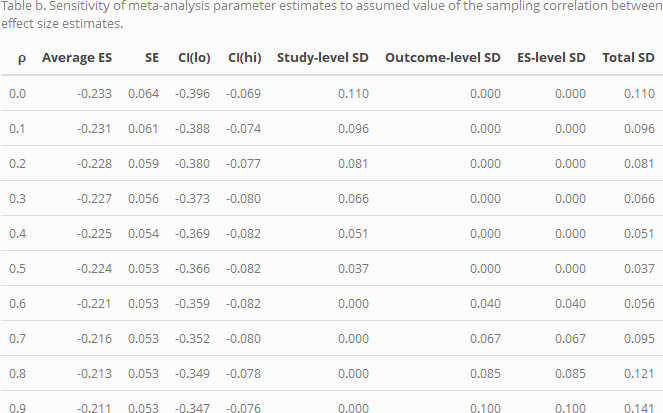

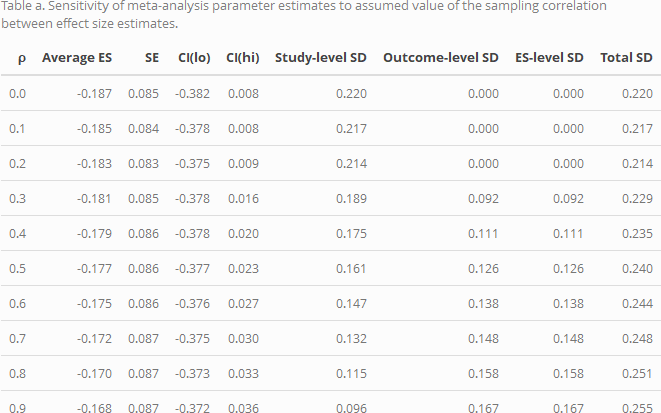


**Figure S3. Funnel plot for EO condition of gender comparisons.** Funnel plot showing the relationships between the gender and postural sway in EO condition of the 10 studies. The standard errors of those SMDs express the precision. The outer dashed lines indicate the triangular region within which 95% of studies are expected to lie in the absence of both biases and heterogeneity. The small-study effect is quantified using Egger’s test.

SMD, Standardized mean difference.


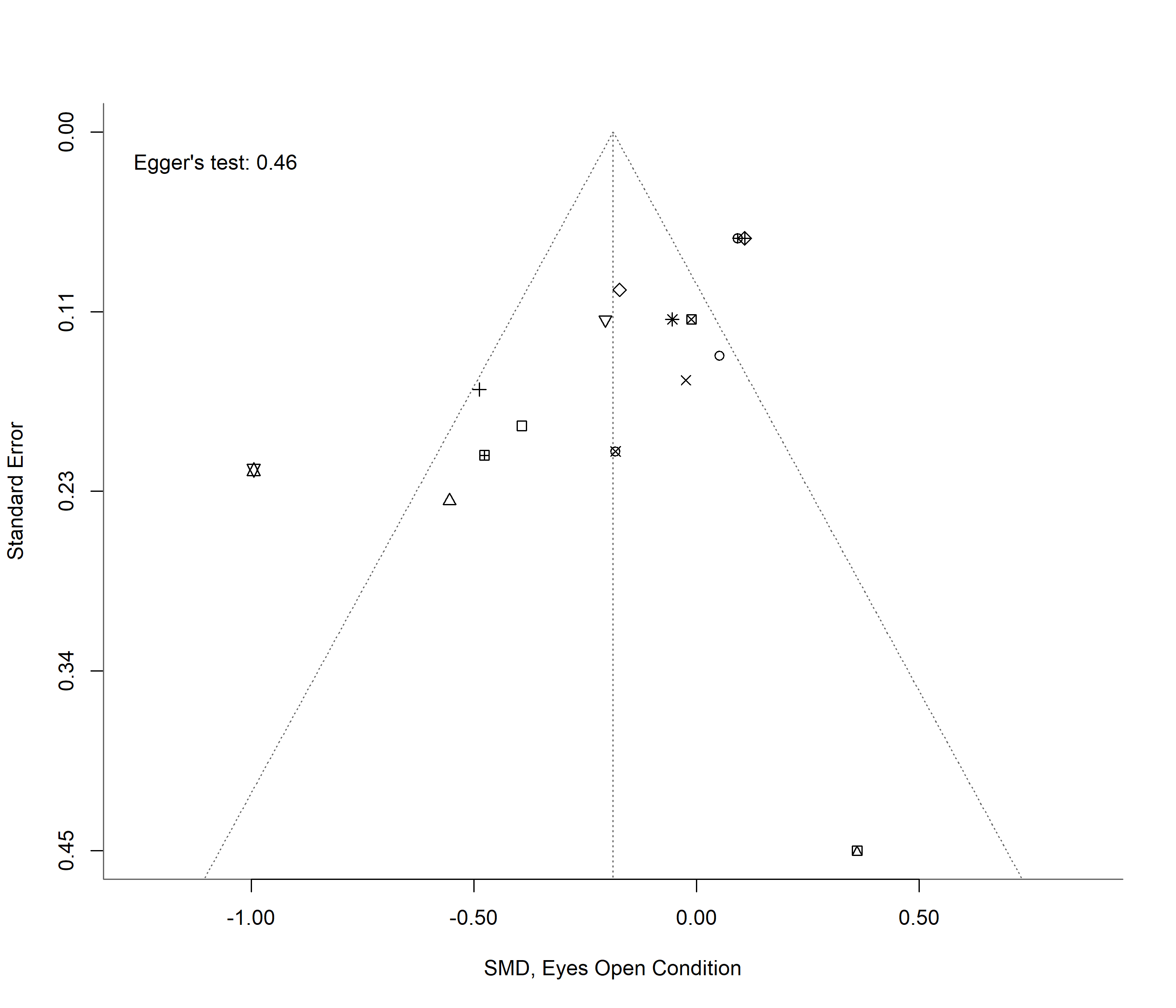


**Table S5. Subgroup analyses for EO condition.**

| **Moderator** | **n of experiments** | **Test for subgroup differences** | **Overall**  **I^2^** | **QE** |
| --- | --- | --- | --- | --- |
| Population Group | 15 | F(2, 7) = 0.046, p = 0.955 | 80.5% | p < 0.001 |
| Region | 15 | F(4, 5) = 0.055, p = 0.993 | 84.5% | p < 0.001 |
| RoB | 15 | F(1, 8) = 0.12, p = 0.741 | 78.1% | p < 0.001 |

**Section C. Frequentist network meta-analysis.**

**Table S6. Distribution of potential effect modifiers in EO and EC conditions.**

| **STUDY INFORMATION** | | | **POPULATION CHARACTERISTICS** | | | | **INTERVENTION** | | | | **Risk of Bias** |
| --- | --- | --- | --- | --- | --- | --- | --- | --- | --- | --- | --- |
| References | Groups | Region | Population Group | Sample size | Females (n) | Males (n) | Posturography Type | Movement Control Type | Tasks | Sway Parameters |  |
| Barozzi et al., 2014 | 6 (n = 20), 7 (n = 43), 8 (n = 38), 9 (n = 27), 10 (n = 45), 11 (n = 29), 12 (n = 41), 13 (n = 35), 14 (n = 11), adults (n = 30) | Europe | Both | 319 | 139 | 180 | Static | Static | mCTSIB | COP_vel, COP_area | High |
| Casselbrant et al., 2010 | 4 (n = 51), 5 (n = 76), 6 (n = 74), 7 (n = 69), 8 (n = 38), 9 (n = 18) | North America | Children | 127 | 57 | 70 | Dynamic | Static | SOT | SOT_ES, SOT_comp | High |
| Eriksen et al, 2023 | 20 - 29 (n= 20), 30 - 39 (n= 20), 40 - 49 (n= 20), 50 - 59 (n= 20), 60 - 69 (n= 20), 70-79 (n= 20)  F, M | Europe | Adults | 120 | 60 | 60 | Dynamic | Static | SOT, MCT | SOT_ES, SOT_comp, SOT_ratios | High |
| Faraldo-Garcia et al., 2016 | 16-20 (n =10), 20 - 29 (n =10), 30 - 39 (n =10), 40 - 49 (n =10), 50 - 59 (n =10), 60 - 69 (n =10), 70-81 (n =10) | Europe | Adults | 70 | 35 | 35 | Dynamic | Static | SOT | SOT_ES | High |
| Henry et al., 2022 | F, M | North America | Both | 253 | 70 | 183 | Dynamic | Static | SOT | SOT_ES, SOT_comp, SOT_ratios | Low |
| Lara et al., 2018 | F, M | Central/South America | Children | 80 | 47 | 33 | Dynamic | Static | SOT | SOT_ES, SOT_comp | Low |
| Letz et al., 1996 | F, M | Europe | Both | 174 | 60 | 114 | Static | Static | EC_FIRM | COP_vel | Low |
| Libardoni et al., 2018 | 8 (n = 33), 9 (n = 33), 10 (n = 33), 11 (n = 33), 12 (n = 33)  F, M | Central/South America | Children | 165 | 82 | 83 | Dynamic | Static | SOT | SOT_ES | High |
| Micarelli et al., 2020 | 4 (n = 10), 5 (n = 10), 6 (n = 10), 7 (n = 10), 8 (n = 10), 9 (n = 10), 10 (n = 10), 11 (n = 10), 12 (n = 10), 13 (n = 10), 14 (n = 10), 15 (n = 10), 16 (n = 10), 17 (n = 10), 18 (n = 10), adults (n = 32) | Europe | Both | 182 | 91 | 91 | Static | Static |  |  |  |
| Mnejja et al., 2022 | 4 (n = 205), 5 (n = 205)  F, M | Africa | Children | 410 | 205 | 205 | Static | Static | mCTSIB | COP_vel | Low |
| Nishino et al., 2021 | 20 - 59 (n = 141), 60 - 69 (n = 82), 70 - 89 (n = 74)  F, M | Central/South America | Adults | 297 | 172 | 125 | Dynamic | Static | Vision_Support | COP_area, COP_velML | Low |
| Patti et al., 2018 | F, M | Europe | Both | 914 | 510 | 404 | Static | Static | EO_FIRM | COP_path, COP_area | Low |
| Shams et al., 2020 | F, M | Asia | Both | 900 | 450 | 450 | Dynamic | Static | SOT | COP_vel, COP_velAP, COP_velML | Low |
| Sinno et al., 2021 | F, M | Asia | Both | 140 | 70 | 70 | Dynamic | Static | SOT | SOT_ES, SOT_comp, SOT_ratios | Low |
| Trueblood et al., 2018 | 20 - 29 (n = 10), 30 - 39 (n = 10), 40 - 49 (n = 10), 50 - 59 (n = 10), 60 - 69 (n = 10) | North America | Adults | 50 | 33 | 17 | Dynamic | Static | SOT | SOT_ES, SOT_comp, SOT_ratios, MCT_comp | High |
| Verbecque et al., 2016 | 3 (n = 24), 4 (n = 27), 5 (n = 28) | Europe | Children | 79 | 39 | 40 | Static | Static | mCTSIB | COP_rangeAP, COP_rangeML, COP_velAP, COP_velML, COP_area, COP_path | Low |

**Figure S4. Subnetworks for different age comparisons for A) EO and B) EC conditions.** Network meta-analysis included 10 studies and revealed two subnetworks, which consisted of 22 treatments, 358 comparisons and 9 study designs. A) The first subnetwork represents adults aged between 20 and 79 years old and is colored in A) light blue and B) dark blue. The second subnetwork represents children aged between 3 and 18 years old and is colored in A) light green and B) dark green.


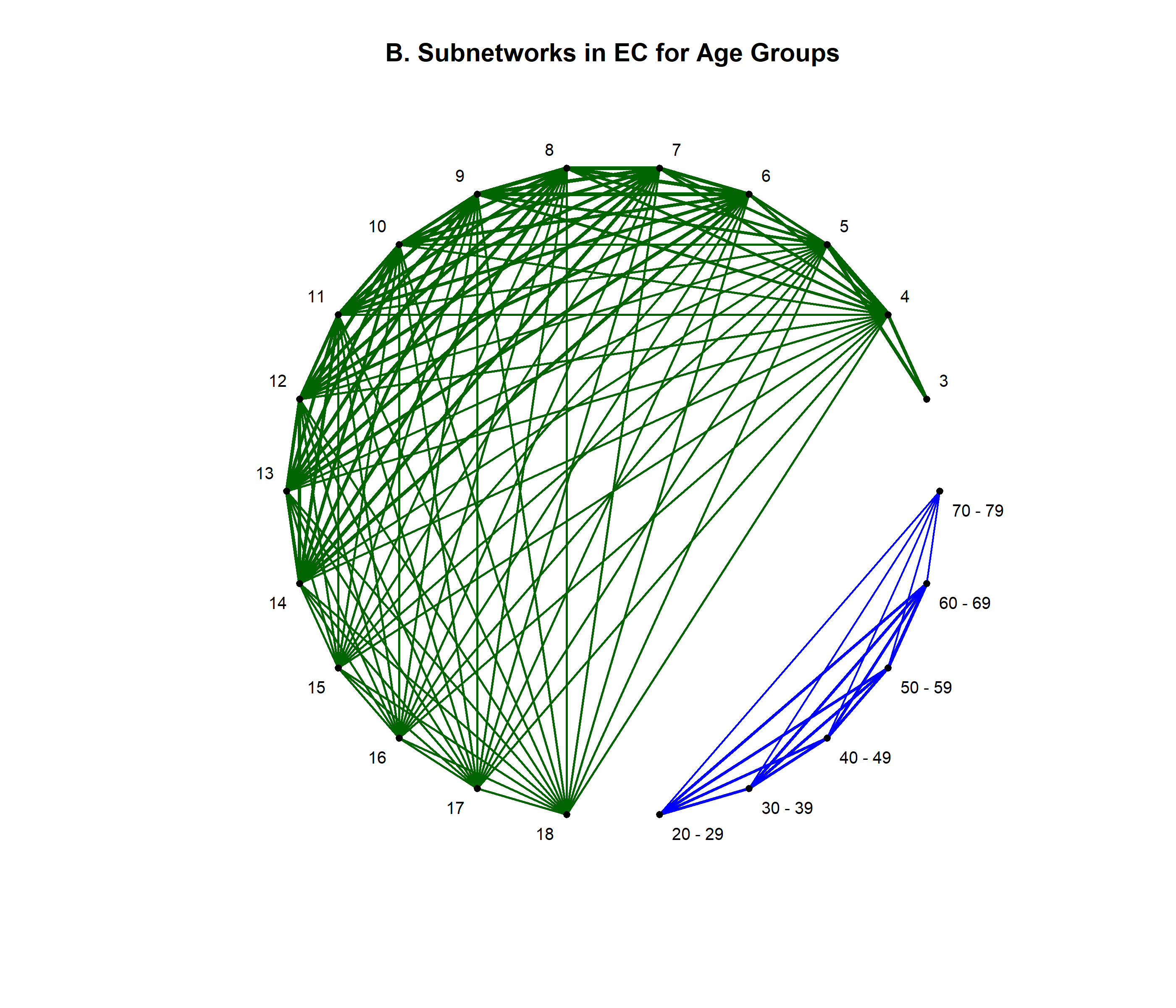

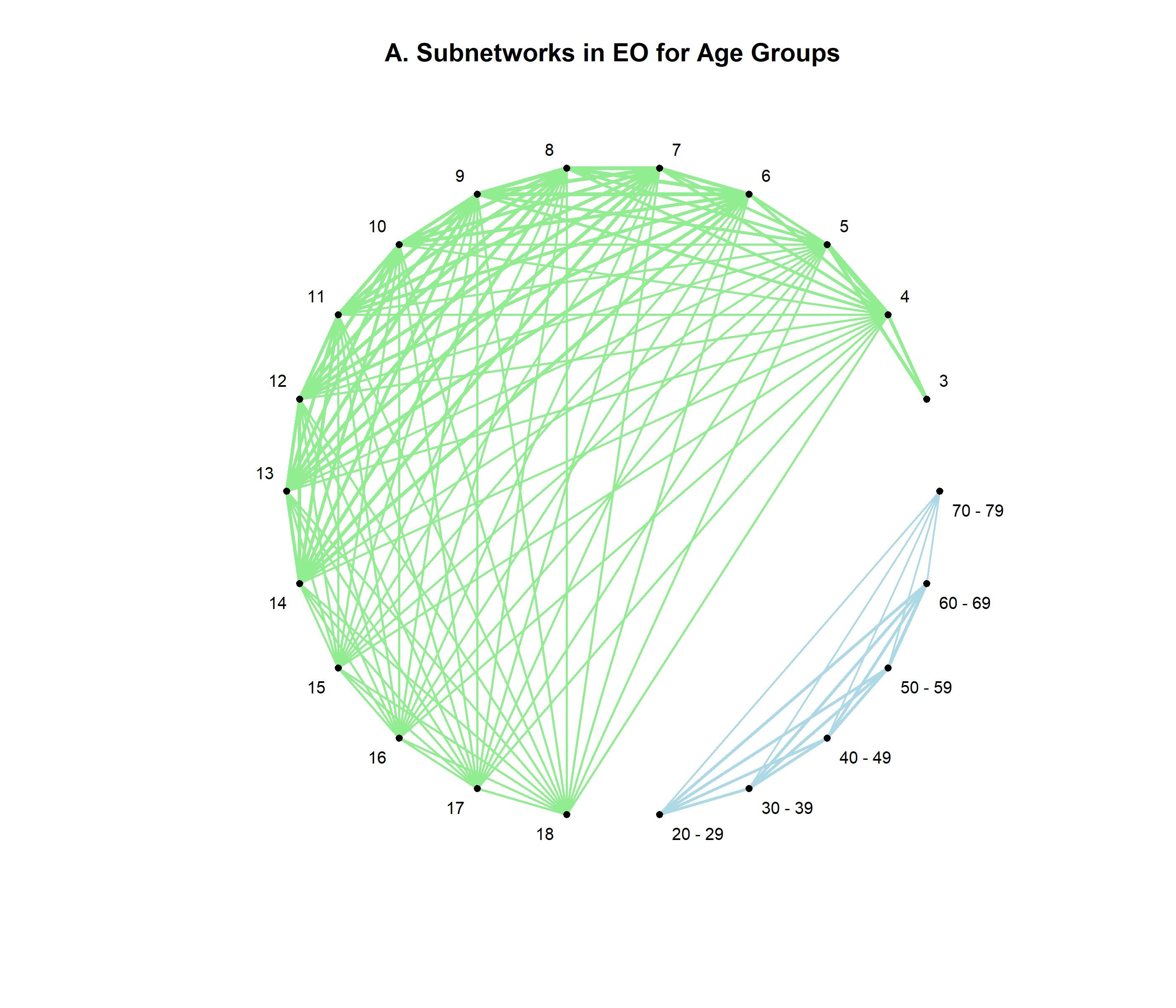


**Table S7. Direct and indirect evidence and network meta-analysis summary table of the first subnetwork for both conditions.**

| **Comparisons** | | **Direct estimate** | **Indirect estimate** | **Network meta-analysis** |  |
| --- | --- | --- | --- | --- | --- |
| **EO CONDITION** | | | | | |
| 70 - 79 | 60 - 69 | 0.06 [-0.56;0.68] | 0.63 [-0.33;1.60] | 0.22 [-0.30;0.75] |  |
| 70 - 79 | 50 - 59 | 0.23 [-0.39;0.85] | 0.24 [-0.73;1.20] | 0.23 [-0.29;0.75] |  |
| 70 - 79 | 40 - 49 | 0.73 [ 0.11;1.36] | 0.00 [-1.24;1.24] | 0.59 [ 0.03;1.15] |  |
| 70 - 79 | 30 - 39 | 0.45 [-0.17;1.07] | 0.86 [-0.39;2.11] | 0.53 [-0.02;1.09] |  |
| 70 - 79 | 20 - 29 | 0.79 [ 0.16;1.42] | 0.15 [-1.09;1.39] | 0.66 [ 0.10;1.22] |  |
| 60 - 69 | 50 - 59 | 0.01 [-0.21;0.22] | . | 0.01 [-0.21;0.22] |  |
| 60 - 69 | 40 - 49 | 0.37 [-0.07;0.81] | 0.34 [-0.58;1.25] | 0.36 [-0.04;0.76] |  |
| 60 - 69 | 30 - 39 | 0.32 [-0.12;0.76] | 0.28 [-0.63;1.18] | 0.31 [-0.09;0.70] |  |
| 60 - 69 | 20 - 29 | 0.44 [ 0.00;0.88] | 0.41 [-0.51;1.32] | 0.44 [ 0.04;0.83] |  |
| 50 - 59 | 40 - 49 | 0.36 [-0.09;0.80] | 0.36 [-0.55;1.26] | 0.36 [-0.04;0.75] |  |
| 50 - 59 | 30 - 39 | 0.31 [-0.13;0.75] | 0.29 [-0.61;1.20] | 0.30 [-0.09;0.70] |  |
| 50 - 59 | 20 - 29 | 0.43 [-0.01;0.87] | 0.43 [-0.48;1.34] | 0.43 [ 0.03;0.83] |  |
| 40 - 49 | 30 - 39 | -0.05 [-0.49;0.38] | . | -0.05 [-0.49;0.39] |  |
| 40 - 49 | 20 - 29 | 0.08 [-0.36;0.51] | . | 0.07 [-0.36;0.51] |  |
| 30 - 39 | 20 - 29 | 0.13 [-0.31;0.57] | . | 0.13 [-0.31;0.57] |  |
| **EC CONDITION** | | | | | |
| 70 - 79 | 60 - 69 | 0.24 [-0.38;0.86] | -0.26 [-1.23;0.72] | 0.09 [-0.43;0.62] |  |
| 70 - 79 | 50 - 59 | 0.14 [-0.48;0.76] | 0.83 [-0.14;1.81] | 0.34 [-0.18;0.86] |  |
| 70 - 79 | 40 - 49 | 0.76 [ 0.13;1.39] | 0.47 [-0.78;1.72] | 0.70 [ 0.14;1.26] |  |
| 70 - 79 | 30 - 39 | 0.85 [ 0.22;1.48] | 0.80 [-0.45;2.05] | 0.84 [ 0.28;1.40] |  |
| 70 - 79 | 20 - 29 | 0.85 [ 0.22;1.48] | 0.85 [-0.40;2.11] | 0.85 [ 0.29;1.42] |  |
| 60 - 69 | 50 - 59 | 0.25 [ 0.03;0.46] | . | 0.25 [ 0.03;0.46] |  |
| 60 - 69 | 40 - 49 | 0.43 [-0.01;0.87] | 1.38 [ 0.47;2.30] | 0.61 [ 0.21;1.00] |  |
| 60 - 69 | 30 - 39 | 0.57 [ 0.13;1.02] | 1.52 [ 0.59;2.45] | 0.75 [ 0.35;1.15] |  |
| 60 - 69 | 20 - 29 | 0.59 [ 0.14;1.03] | 1.53 [ 0.60;2.46] | 0.76 [ 0.36;1.16] |  |
| 50 - 59 | 40 - 49 | 0.55 [ 0.11;0.99] | -0.44 [-1.35;0.46] | 0.36 [-0.04;0.76] |  |
| 50 - 59 | 30 - 39 | 0.69 [ 0.25;1.14] | -0.30 [-1.21;0.61] | 0.50 [ 0.10;0.90] |  |
| 50 - 59 | 20 - 29 | 0.70 [ 0.26;1.15] | -0.28 [-1.20;0.63] | 0.51 [ 0.11;0.91] |  |
| 40 - 49 | 30 - 39 | 0.15 [-0.29;0.58] | . | 0.14 [-0.30;0.58] |  |
| 40 - 49 | 20 - 29 | 0.16 [-0.28;0.60] | . | 0.15 [-0.29;0.59] |  |
| 30 - 39 | 20 - 29 | 0.01 [-0.43;0.45] | . | 0.01 [-0.43;0.45] |  |

**Table S8. Direct and indirect evidence and network meta-analysis summary table of the second subnetwork for both conditions.**

| **Comparisons** | | **Direct estimate** | **Indirect estimate** | **Network meta-analysis** |
| --- | --- | --- | --- | --- |
| **EO CONDITION** | | | | |
| 3 | 4 | 0.11 [-0.37; 0.60] | -2.76 [ -4.40;-1.13] | -0.12 [-0.59;0.35] |
| 3 | 5 | -0.58 [-1.07;-0.09] | 2.29 [ 0.66; 3.92] | -0.35 [-0.81;0.12] |
| 3 | 6 | . | 0.08 [ -0.76; 0.91] | 0.08 [-0.76;0.91] |
| 3 | 7 | . | 0.43 [ -0.40; 1.26] | 0.43 [-0.40;1.26] |
| 3 | 8 | . | 0.75 [ -0.08; 1.59] | 0.75 [-0.08;1.59] |
| 3 | 9 | . | 1.01 [ 0.16; 1.87] | 1.01 [ 0.16;1.87] |
| 3 | 10 | . | 0.98 [ 0.03; 1.93] | 0.98 [ 0.03;1.93] |
| 3 | 11 | . | 1.16 [ 0.20; 2.12] | 1.16 [ 0.20;2.12] |
| 3 | 12 | . | 1.33 [ 0.38; 2.28] | 1.33 [ 0.38;2.28] |
| 3 | 13 | . | 1.45 [ 0.50; 2.41] | 1.45 [ 0.50;2.41] |
| 3 | 14 | . | 1.43 [ 0.41; 2.45] | 1.43 [ 0.41;2.45] |
| 4 | 5 | -0.23 [-0.62; 0.17] | . | -0.23 [-0.62;0.17] |
| 4 | 6 | 0.88 [-0.12; 1.89] | -0.67 [ -1.80; 0.46] | 0.20 [-0.55;0.95] |
| 4 | 7 | 1.03 [ 0.03; 2.04] | -0.04 [ -1.15; 1.07] | 0.55 [-0.20;1.29] |
| 4 | 8 | 1.18 [ 0.15; 2.21] | 0.52 [ -0.58; 1.63] | 0.87 [ 0.12;1.63] |
| 4 | 9 | 1.62 [ 0.54; 2.71] | 0.63 [ -0.47; 1.73] | 1.13 [ 0.36;1.90] |
| 4 | 10 | . | 1.10 [ 0.23; 1.97] | 1.10 [ 0.23;1.97] |
| 4 | 11 | . | 1.28 [ 0.39; 2.16] | 1.28 [ 0.39;2.16] |
| 4 | 12 | . | 1.45 [ 0.58; 2.33] | 1.45 [ 0.58;2.33] |
| 4 | 13 | . | 1.57 [ 0.69; 2.45] | 1.57 [ 0.69;2.45] |
| 4 | 14 | . | 1.55 [ 0.60; 2.50] | 1.55 [ 0.60;2.50] |
| 5 | 6 | 0.15 [-0.84; 1.14] | 0.79 [ -0.35; 1.93] | 0.42 [-0.32;1.17] |
| 5 | 7 | 0.29 [-0.70; 1.29] | 1.38 [ 0.26; 2.50] | 0.77 [ 0.03;1.51] |
| 5 | 8 | 0.44 [-0.57; 1.46] | 1.89 [ 0.78; 3.01] | 1.10 [ 0.35;1.85] |
| 5 | 9 | 0.88 [-0.18; 1.95] | 1.87 [ 0.76; 2.97] | 1.36 [ 0.59;2.13] |
| 5 | 10 | . | 1.33 [ 0.46; 2.20] | 1.33 [ 0.46;2.20] |
| 5 | 11 | . | 1.50 [ 0.62; 2.39] | 1.50 [ 0.62;2.39] |
| 5 | 12 | . | 1.68 [ 0.81; 2.55] | 1.68 [ 0.81;2.55] |
| 5 | 13 | . | 1.80 [ 0.92; 2.68] | 1.80 [ 0.92;2.68] |
| 5 | 14 | . | 1.78 [ 0.83; 2.72] | 1.78 [ 0.83;2.72] |
| 6 | 7 | 0.36 [-0.25; 0.96] | -6.95 [-25.87;11.98] | 0.35 [-0.25;0.95] |
| 6 | 8 | 0.68 [ 0.07; 1.29] | -0.75 [-14.93;13.43] | 0.68 [ 0.07;1.29] |
| 6 | 9 | 0.95 [ 0.32; 1.58] | -3.12 [-12.89; 6.65] | 0.93 [ 0.31;1.56] |
| 6 | 10 | 1.03 [ 0.27; 1.79] | 0.24 [ -1.50; 1.98] | 0.90 [ 0.20;1.60] |
| 6 | 11 | 1.20 [ 0.43; 1.98] | 0.39 [ -1.43; 2.21] | 1.08 [ 0.36;1.79] |
| 6 | 12 | 1.38 [ 0.62; 2.15] | 0.58 [ -1.18; 2.34] | 1.26 [ 0.55;1.96] |
| 6 | 13 | 1.50 [ 0.73; 2.28] | 0.69 [ -1.10; 2.47] | 1.38 [ 0.67;2.08] |
| 6 | 14 | 1.48 [ 0.64; 2.33] | 0.51 [ -1.67; 2.69] | 1.35 [ 0.57;2.14] |
| 7 | 8 | 0.33 [-0.27; 0.92] | . | 0.33 [-0.26;0.92] |
| 7 | 9 | 0.59 [-0.02; 1.20] | -1.08 [-21.27;19.11] | 0.59 [-0.03;1.20] |
| 7 | 10 | 0.55 [-0.18; 1.27] | 0.58 [ -1.32; 2.48] | 0.55 [-0.12;1.23] |
| 7 | 11 | 0.73 [-0.02; 1.47] | 0.76 [ -1.23; 2.75] | 0.73 [ 0.03;1.42] |
| 7 | 12 | 0.90 [ 0.18; 1.63] | 0.93 [ -0.99; 2.84] | 0.91 [ 0.23;1.59] |
| 7 | 13 | 1.03 [ 0.29; 1.76] | 1.04 [ -0.91; 2.99] | 1.03 [ 0.34;1.72] |
| 7 | 14 | 1.00 [ 0.19; 1.81] | 1.03 [ -1.39; 3.44] | 1.00 [ 0.23;1.77] |
| 8 | 9 | 0.25 [-0.36; 0.87] | . | 0.26 [-0.36;0.87] |
| 8 | 10 | 0.13 [-0.60; 0.86] | 0.89 [ -1.03; 2.81] | 0.23 [-0.46;0.91] |
| 8 | 11 | 0.31 [-0.44; 1.05] | 1.10 [ -0.92; 3.11] | 0.40 [-0.30;1.10] |
| 8 | 12 | 0.48 [-0.25; 1.22] | 1.24 [ -0.70; 3.19] | 0.58 [-0.11;1.26] |
| 8 | 13 | 0.61 [-0.13; 1.34] | 1.37 [ -0.60; 3.35] | 0.70 [ 0.01;1.39] |
| 8 | 14 | 0.58 [-0.23; 1.40] | 1.52 [ -0.92; 3.96] | 0.68 [-0.10;1.45] |
| 9 | 10 | -0.04 [-0.78; 0.71] | -0.00 [ -1.96; 1.95] | -0.03 [-0.73;0.66] |
| 9 | 11 | 0.14 [-0.62; 0.90] | 0.17 [ -1.88; 2.21] | 0.14 [-0.57;0.86] |
| 9 | 12 | 0.32 [-0.43; 1.06] | 0.34 [ -1.63; 2.31] | 0.32 [-0.38;1.02] |
| 9 | 13 | 0.44 [-0.31; 1.19] | 0.46 [ -1.55; 2.47] | 0.44 [-0.26;1.15] |
| 9 | 14 | 0.42 [-0.41; 1.24] | 0.44 [ -2.02; 2.91] | 0.42 [-0.36;1.20] |
| 10 | 11 | 0.18 [-0.56; 0.92] | . | 0.18 [-0.56;0.92] |
| 10 | 12 | 0.36 [-0.37; 1.08] | . | 0.35 [-0.37;1.08] |
| 10 | 13 | 0.48 [-0.26; 1.21] | . | 0.47 [-0.26;1.21] |
| 10 | 14 | 0.45 [-0.36; 1.26] | . | 0.45 [-0.36;1.26] |
| 11 | 12 | 0.18 [-0.56; 0.92] | . | 0.18 [-0.56;0.92] |
| 11 | 13 | 0.30 [-0.45; 1.05] | . | 0.30 [-0.45;1.05] |
| 11 | 14 | 0.27 [-0.55; 1.10] | . | 0.27 [-0.55;1.10] |
| 12 | 13 | 0.12 [-0.61; 0.85] | . | 0.12 [-0.61;0.85] |
| 12 | 14 | 0.10 [-0.71; 0.91] | . | 0.10 [-0.71;0.91] |
| 13 | 14 | -0.02 [-0.84; 0.79] | . | -0.02 [-0.84;0.79] |
| **EC CONDITION** | | | | |
| 3 | 4 | -0.02 [-0.31; 0.27] | -1.59 [-2.41;-0.76] | -0.19 [-0.46; 0.09] |
| 3 | 5 | -0.47 [-0.76;-0.17] | 1.12 [ 0.29; 1.95] | -0.29 [-0.57;-0.02] |
| 3 | 6 | . | 0.20 [-0.24; 0.65] | 0.20 [-0.24; 0.65] |
| 3 | 7 | . | 0.51 [ 0.07; 0.96] | 0.51 [ 0.07; 0.96] |
| 3 | 8 | . | 0.72 [ 0.26; 1.18] | 0.72 [ 0.26; 1.18] |
| 3 | 9 | . | 0.86 [ 0.37; 1.34] | 0.86 [ 0.37; 1.34] |
| 3 | 10 | . | 0.65 [ 0.13; 1.16] | 0.65 [ 0.13; 1.16] |
| 3 | 11 | . | 0.99 [ 0.45; 1.52] | 0.99 [ 0.45; 1.52] |
| 3 | 12 | . | 1.06 [ 0.54; 1.58] | 1.06 [ 0.54; 1.58] |
| 3 | 13 | . | 1.01 [ 0.48; 1.53] | 1.01 [ 0.48; 1.53] |
| 3 | 14 | . | 1.26 [ 0.63; 1.89] | 1.26 [ 0.63; 1.89] |
| 4 | 5 | -0.10 [-0.31; 0.11] | . | -0.10 [-0.31; 0.11] |
| 4 | 6 | 0.71 [ 0.21; 1.21] | -0.08 [-0.69; 0.53] | 0.39 [ 0.01; 0.78] |
| 4 | 7 | 0.83 [ 0.32; 1.33] | 0.53 [-0.05; 1.12] | 0.70 [ 0.32; 1.08] |
| 4 | 8 | 1.19 [ 0.63; 1.74] | 0.60 [ 0.02; 1.18] | 0.91 [ 0.51; 1.31] |
| 4 | 9 | 1.42 [ 0.77; 2.07] | 0.75 [ 0.17; 1.33] | 1.05 [ 0.62; 1.48] |
| 4 | 10 | . | 0.84 [ 0.38; 1.30] | 0.84 [ 0.38; 1.30] |
| 4 | 11 | . | 1.18 [ 0.69; 1.66] | 1.18 [ 0.69; 1.66] |
| 4 | 12 | . | 1.25 [ 0.78; 1.72] | 1.25 [ 0.78; 1.72] |
| 4 | 13 | . | 1.19 [ 0.72; 1.67] | 1.19 [ 0.72; 1.67] |
| 4 | 14 | . | 1.45 [ 0.86; 2.04] | 1.45 [ 0.86; 2.04] |
| 5 | 6 | 0.36 [-0.12; 0.83] | 0.76 [ 0.11; 1.40] | 0.50 [ 0.12; 0.88] |
| 5 | 7 | 0.47 [-0.00; 0.95] | 1.34 [ 0.74; 1.95] | 0.81 [ 0.43; 1.18] |
| 5 | 8 | 0.83 [ 0.30; 1.35] | 1.24 [ 0.65; 1.83] | 1.01 [ 0.62; 1.40] |
| 5 | 9 | 1.07 [ 0.44; 1.69] | 1.22 [ 0.64; 1.80] | 1.15 [ 0.72; 1.57] |
| 5 | 10 | . | 0.94 [ 0.48; 1.39] | 0.94 [ 0.48; 1.39] |
| 5 | 11 | . | 1.28 [ 0.80; 1.76] | 1.28 [ 0.80; 1.76] |
| 5 | 12 | . | 1.35 [ 0.89; 1.81] | 1.35 [ 0.89; 1.81] |
| 5 | 13 | . | 1.30 [ 0.83; 1.77] | 1.30 [ 0.83; 1.77] |
| 5 | 14 | . | 1.56 [ 0.97; 2.14] | 1.56 [ 0.97; 2.14] |
| 6 | 7 | 0.30 [-0.03; 0.63] | 1.28 [-2.38; 4.94] | 0.31 [-0.02; 0.64] |
| 6 | 8 | 0.53 [ 0.19; 0.87] | -0.39 [-2.84; 2.06] | 0.51 [ 0.17; 0.85] |
| 6 | 9 | 0.69 [ 0.31; 1.07] | -0.29 [-2.14; 1.57] | 0.65 [ 0.28; 1.02] |
| 6 | 10 | 0.51 [ 0.07; 0.96] | 0.23 [-0.54; 1.00] | 0.44 [ 0.06; 0.83] |
| 6 | 11 | 0.86 [ 0.38; 1.33] | 0.54 [-0.34; 1.42] | 0.78 [ 0.37; 1.20] |
| 6 | 12 | 0.93 [ 0.48; 1.38] | 0.64 [-0.16; 1.43] | 0.86 [ 0.46; 1.25] |
| 6 | 13 | 0.87 [ 0.41; 1.33] | 0.57 [-0.26; 1.40] | 0.80 [ 0.40; 1.20] |
| 6 | 14 | 1.13 [ 0.55; 1.71] | 0.66 [-0.71; 2.02] | 1.06 [ 0.53; 1.59] |
| 7 | 8 | 0.20 [-0.12; 0.51] | 3.82 [-4.19;11.82] | 0.20 [-0.11; 0.52] |
| 7 | 9 | 0.33 [-0.01; 0.68] | 1.58 [-2.31; 5.47] | 0.34 [-0.00; 0.69] |
| 7 | 10 | 0.05 [-0.33; 0.43] | 0.67 [-0.30; 1.64] | 0.13 [-0.22; 0.49] |
| 7 | 11 | 0.39 [-0.02; 0.81] | 1.11 [-0.04; 2.26] | 0.47 [ 0.09; 0.86] |
| 7 | 12 | 0.46 [ 0.07; 0.86] | 1.10 [ 0.09; 2.11] | 0.55 [ 0.18; 0.91] |
| 7 | 13 | 0.41 [ 0.01; 0.81] | 1.08 [ 0.02; 2.15] | 0.49 [ 0.12; 0.87] |
| 7 | 14 | 0.67 [ 0.14; 1.20] | 1.84 [-0.09; 3.77] | 0.75 [ 0.24; 1.26] |
| 8 | 9 | 0.15 [-0.21; 0.50] | -1.92 [-8.77; 4.93] | 0.14 [-0.22; 0.50] |
| 8 | 10 | -0.06 [-0.45; 0.33] | -0.14 [-1.15; 0.87] | -0.07 [-0.44; 0.29] |
| 8 | 11 | 0.28 [-0.14; 0.70] | 0.18 [-1.00; 1.37] | 0.27 [-0.13; 0.67] |
| 8 | 12 | 0.36 [-0.04; 0.75] | 0.27 [-0.78; 1.31] | 0.34 [-0.03; 0.72] |
| 8 | 13 | 0.30 [-0.11; 0.71] | 0.21 [-0.89; 1.31] | 0.29 [-0.09; 0.67] |
| 8 | 14 | 0.56 [ 0.02; 1.09] | 0.39 [-1.57; 2.36] | 0.55 [ 0.03; 1.06] |
| 9 | 10 | -0.17 [-0.58; 0.25] | -0.50 [-1.57; 0.58] | -0.21 [-0.60; 0.18] |
| 9 | 11 | 0.17 [-0.27; 0.62] | -0.21 [-1.44; 1.03] | 0.13 [-0.29; 0.55] |
| 9 | 12 | 0.25 [-0.17; 0.67] | -0.10 [-1.20; 1.01] | 0.20 [-0.19; 0.60] |
| 9 | 13 | 0.19 [-0.24; 0.62] | -0.17 [-1.32; 0.99] | 0.15 [-0.26; 0.55] |
| 9 | 14 | 0.45 [-0.10; 1.00] | -0.15 [-2.11; 1.82] | 0.41 [-0.13; 0.94] |
| 10 | 11 | 0.34 [-0.07; 0.75] | . | 0.34 [-0.07; 0.75] |
| 10 | 12 | 0.41 [ 0.03; 0.80] | . | 0.41 [ 0.03; 0.80] |
| 10 | 13 | 0.36 [-0.04; 0.76] | . | 0.36 [-0.04; 0.76] |
| 10 | 14 | 0.62 [ 0.09; 1.14] | . | 0.62 [ 0.09; 1.14] |
| 11 | 12 | 0.07 [-0.34; 0.49] | . | 0.07 [-0.34; 0.49] |
| 11 | 13 | 0.02 [-0.41; 0.44] | . | 0.02 [-0.41; 0.44] |
| 11 | 14 | 0.28 [-0.27; 0.82] | . | 0.28 [-0.27; 0.82] |
| 12 | 13 | -0.06 [-0.46; 0.35] | . | -0.06 [-0.46; 0.35] |
| 12 | 14 | 0.20 [-0.33; 0.73] | . | 0.20 [-0.33; 0.73] |
| 13 | 14 | 0.26 [-0.28; 0.80] | . | 0.26 [-0.28; 0.80] |

**Table S9. The analysis of Separate indirect from direct evidence (SIDE) (back-calculation method).**

1. **First subnetwork for EO condition.**


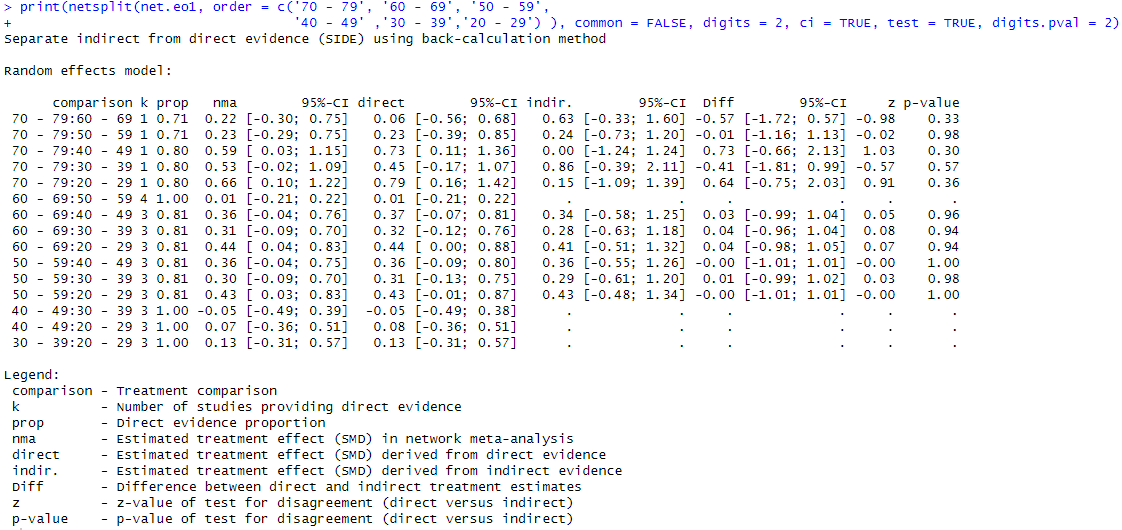


1. **First subnetwork for EC condition.**


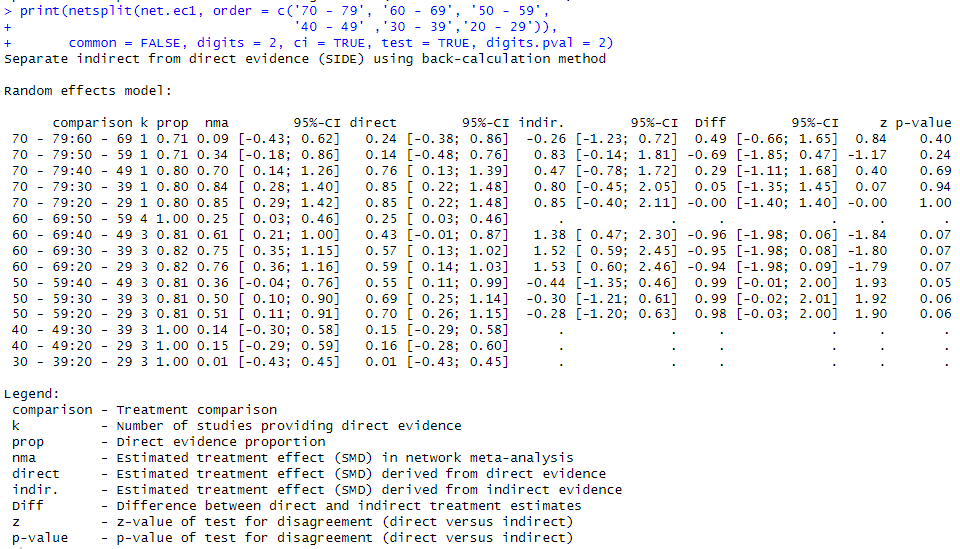


1. **Second subnetwork for EO condition.**


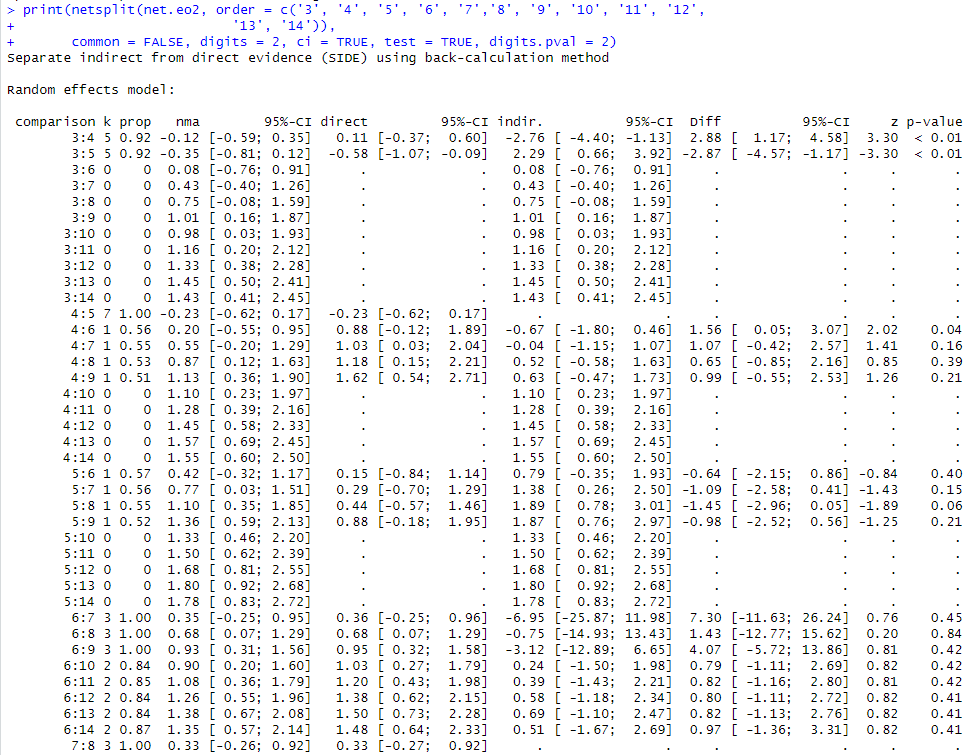

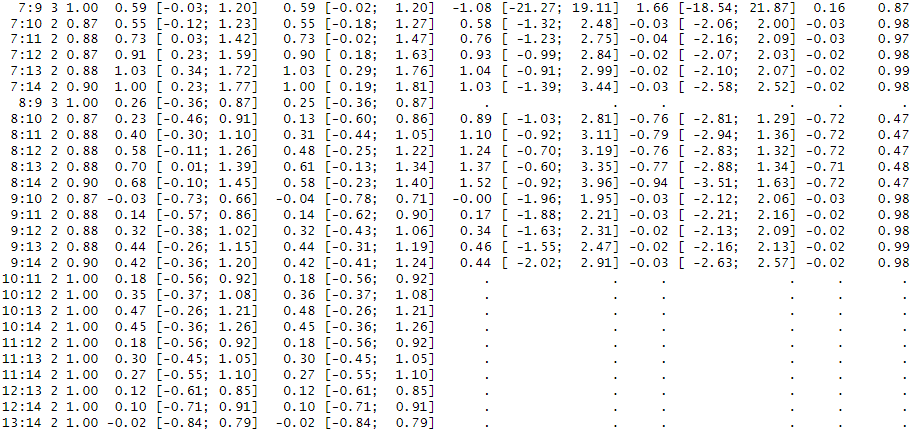


1. **Second subnetwork for EC condition.**


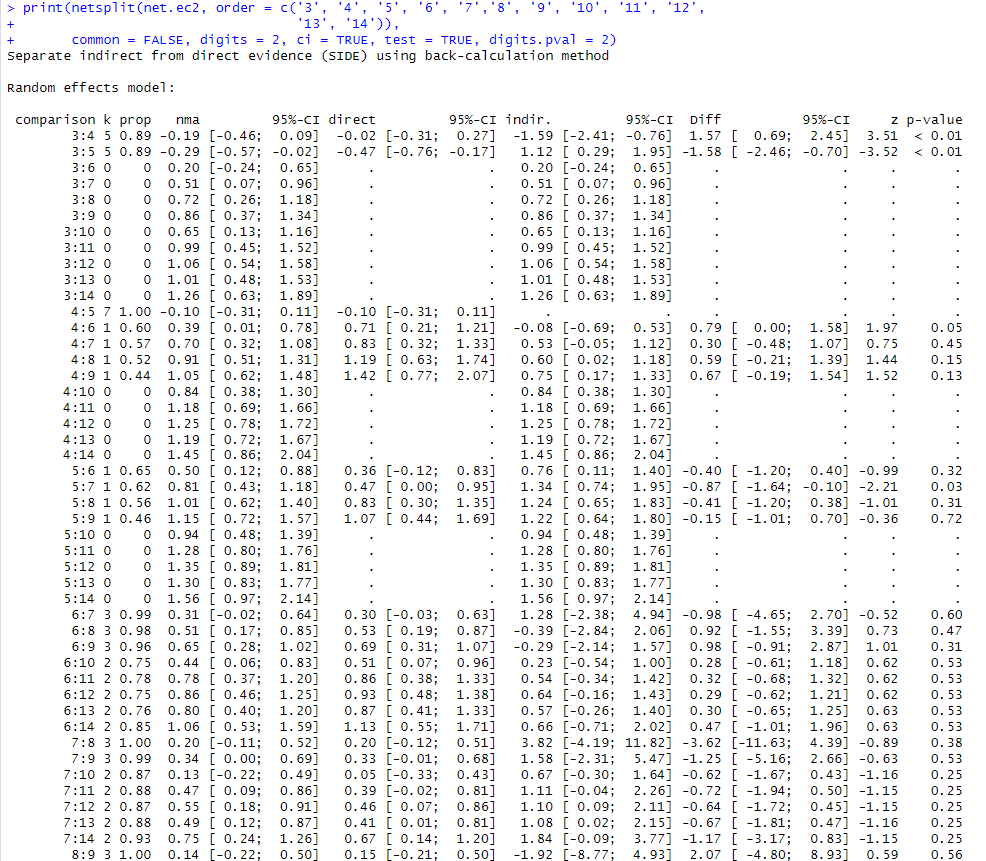

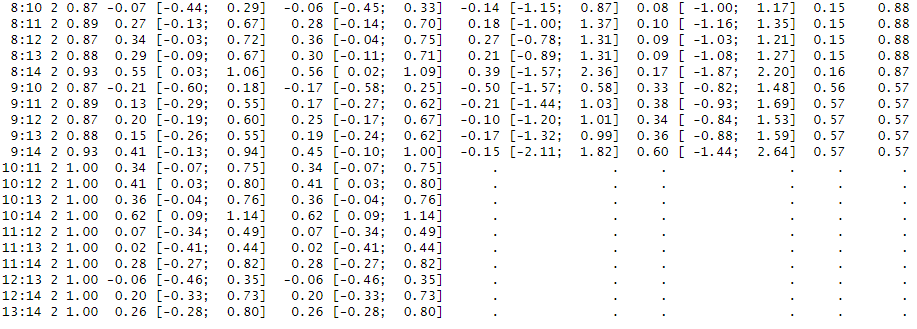


**Table S10. CINeMA grading of the certainty of the evidence.**
A) Certainty of the evidence of the first subnetwork for EO condition.

| **Comparison** | **Number of studies** | **Within-study bias** | **Reporting bias** | **Indirectness** | **Imprecision** | **Heterogeneity** | **Incoherence** | **Confidence rating** | **Reason(s) for downgrading** |
| --- | --- | --- | --- | --- | --- | --- | --- | --- | --- |
| 20 - 29:30 - 39 | 3 | Major concerns | Some concerns | No concerns | Major concerns | No concerns | No concerns | Very low | ["Within-study bias","Reporting bias","Imprecision"] |
| 20 - 29:40 - 49 | 3 | Major concerns | Some concerns | No concerns | Major concerns | No concerns | No concerns | Very low | ["Within-study bias","Reporting bias","Imprecision"] |
| 20 - 29:50 - 59 | 3 | Major concerns | Some concerns | No concerns | No concerns | Some concerns | No concerns | Very low | ["Within-study bias","Reporting bias","Heterogeneity"] |
| 20 - 29:60 - 69 | 3 | Major concerns | Some concerns | No concerns | No concerns | No concerns | No concerns | Low | ["Within-study bias","Reporting bias"] |
| 20 - 29:70 - 79 | 1 | Major concerns | Some concerns | No concerns | No concerns | No concerns | No concerns | Low | ["Within-study bias","Reporting bias"] |
| 30 - 39:40 - 49 | 3 | Major concerns | Some concerns | No concerns | Major concerns | No concerns | No concerns | Very low | ["Within-study bias","Reporting bias","Imprecision"] |
| 30 - 39:50 - 59 | 3 | Major concerns | Some concerns | No concerns | No concerns | Some concerns | No concerns | Very low | ["Within-study bias","Reporting bias","Heterogeneity"] |
| 30 - 39:60 - 69 | 3 | Major concerns | Some concerns | No concerns | No concerns | No concerns | No concerns | Low | ["Within-study bias","Reporting bias"] |
| 30 - 39:70 - 79 | 1 | Major concerns | Some concerns | No concerns | No concerns | No concerns | No concerns | Low | ["Within-study bias","Reporting bias"] |
| 40 - 49:50 - 59 | 3 | Major concerns | Some concerns | No concerns | Some concerns | No concerns | No concerns | Very low | ["Within-study bias","Reporting bias","Imprecision"] |
| 40 - 49:60 - 69 | 3 | Major concerns | Some concerns | No concerns | No concerns | No concerns | No concerns | Low | ["Within-study bias","Reporting bias"] |
| 40 - 49:70 - 79 | 1 | Major concerns | Some concerns | No concerns | No concerns | No concerns | No concerns | Low | ["Within-study bias","Reporting bias"] |
| 50 - 59:60 - 69 | 4 | Some concerns | Some concerns | No concerns | No concerns | Some concerns | No concerns | Very low | ["Within-study bias","Reporting bias","Heterogeneity"] |
| 50 - 59:70 - 79 | 1 | Major concerns | Some concerns | No concerns | Some concerns | Some concerns | No concerns | Very low | ["Within-study bias","Reporting bias","Imprecision","Heterogeneity"] |
| 60 - 69:70 - 79 | 1 | Major concerns | Some concerns | No concerns | Major concerns | No concerns | No concerns | Very low | ["Within-study bias","Reporting bias","Imprecision"] |

B) Certainty of the evidence of the first subnetwork for EC condition.

| **Comparison** | **Number of studies** | **Within-study bias** | **Reporting bias** | **Indirectness** | **Imprecision** | **Heterogeneity** | **Incoherence** | **Confidence rating** | **Reason(s) for downgrading** |
| --- | --- | --- | --- | --- | --- | --- | --- | --- | --- |
| 20 - 29:30 - 39 | 3 | Major concerns | Some concerns | No concerns | Major concerns | No concerns | No concerns | Very low | ["Within-study bias","Reporting bias","Imprecision"] |
| 20 - 29:40 - 49 | 3 | Major concerns | Some concerns | No concerns | Major concerns | No concerns | No concerns | Very low | ["Within-study bias","Reporting bias","Imprecision"] |
| 20 - 29:50 - 59 | 3 | Major concerns | Some concerns | No concerns | No concerns | Some concerns | Some concerns | Very low | ["Within-study bias","Reporting bias","Heterogeneity","Incoherence"] |
| 20 - 29:60 - 69 | 3 | Major concerns | Some concerns | No concerns | No concerns | No concerns | Some concerns | Very low | ["Within-study bias","Reporting bias","Incoherence"] |
| 20 - 29:70 - 79 | 1 | Major concerns | Some concerns | No concerns | No concerns | No concerns | No concerns | Low | ["Within-study bias","Reporting bias"] |
| 30 - 39:40 - 49 | 3 | Major concerns | Some concerns | No concerns | Major concerns | No concerns | No concerns | Very low | ["Within-study bias","Reporting bias","Imprecision"] |
| 30 - 39:50 - 59 | 3 | Major concerns | Some concerns | No concerns | No concerns | Some concerns | Some concerns | Very low | ["Within-study bias","Reporting bias","Heterogeneity","Incoherence"] |
| 30 - 39:60 - 69 | 3 | Major concerns | Some concerns | No concerns | No concerns | No concerns | Some concerns | Very low | ["Within-study bias","Reporting bias","Incoherence"] |
| 30 - 39:70 - 79 | 1 | Major concerns | Some concerns | No concerns | No concerns | No concerns | No concerns | Low | ["Within-study bias","Reporting bias"] |
| 40 - 49:50 - 59 | 3 | Major concerns | Some concerns | No concerns | Some concerns | No concerns | Some concerns | Very low | ["Within-study bias","Reporting bias","Imprecision","Incoherence"] |
| 40 - 49:60 - 69 | 3 | Major concerns | Some concerns | No concerns | No concerns | No concerns | Some concerns | Very low | ["Within-study bias","Reporting bias","Incoherence"] |
| 40 - 49:70 - 79 | 1 | Major concerns | Some concerns | No concerns | No concerns | No concerns | No concerns | Low | ["Within-study bias","Reporting bias"] |
| 50 - 59:60 - 69 | 4 | Some concerns | Some concerns | No concerns | No concerns | Some concerns | No concerns | Very low | ["Within-study bias","Reporting bias","Heterogeneity"] |
| 50 - 59:70 - 79 | 1 | Major concerns | Some concerns | No concerns | Some concerns | Some concerns | No concerns | Very low | ["Within-study bias","Reporting bias","Imprecision","Heterogeneity"] |
| 60 - 69:70 - 79 | 1 | Major concerns | Some concerns | No concerns | Major concerns | No concerns | No concerns | Very low | ["Within-study bias","Reporting bias","Imprecision"] |

C) Certainty of the evidence of the second subnetwork for EO condition.

| **Comparison** | **Number of studies** | **Within-study bias** | **Reporting bias** | **Indirectness** | **Imprecision** | **Heterogeneity** | **Incoherence** | **Confidence rating** | **Reason(s) for downgrading** |
| --- | --- | --- | --- | --- | --- | --- | --- | --- | --- |
| 03:04 | 5 | No concerns | Low risk | No concerns | No concerns | Some concerns | Major concerns | Low | ["Heterogeneity","Incoherence"] |
| 03:05 | 5 | No concerns | Low risk | No concerns | No concerns | Some concerns | Major concerns | Low | ["Heterogeneity","Incoherence"] |
| 04:05 | 7 | Some concerns | Low risk | No concerns | No concerns | Some concerns | Major concerns | Very low | ["Within-study bias","Heterogeneity","Incoherence"] |
| 04:06 | 1 | Major concerns | Low risk | No concerns | No concerns | Major concerns | Major concerns | Very low | ["Within-study bias","Heterogeneity","Incoherence"] |
| 04:07 | 1 | Major concerns | Low risk | No concerns | Some concerns | No concerns | No concerns | Low | ["Within-study bias","Imprecision"] |
| 04:08 | 1 | Major concerns | Low risk | No concerns | No concerns | Some concerns | No concerns | Low | ["Within-study bias","Heterogeneity"] |
| 04:09 | 1 | Major concerns | Low risk | No concerns | No concerns | Some concerns | No concerns | Low | ["Within-study bias","Heterogeneity"] |
| 05:06 | 1 | Major concerns | Low risk | No concerns | Some concerns | No concerns | No concerns | Low | ["Within-study bias","Imprecision"] |
| 05:07 | 1 | Major concerns | Low risk | No concerns | No concerns | Some concerns | Major concerns | Very low | ["Within-study bias","Heterogeneity","Incoherence"] |
| 05:08 | 1 | Major concerns | Low risk | No concerns | No concerns | Some concerns | No concerns | Low | ["Within-study bias","Heterogeneity"] |
| 05:09 | 1 | Major concerns | Low risk | No concerns | No concerns | No concerns | No concerns | Moderate | ["Within-study bias"] |
| 06:07 | 3 | Major concerns | Low risk | No concerns | No concerns | Some concerns | No concerns | Low | ["Within-study bias","Heterogeneity"] |
| 06:08 | 3 | Major concerns | Low risk | No concerns | No concerns | Some concerns | No concerns | Low | ["Within-study bias","Heterogeneity"] |
| 06:09 | 3 | Major concerns | Low risk | No concerns | No concerns | Some concerns | No concerns | Low | ["Within-study bias","Heterogeneity"] |
| 07:08 | 3 | Major concerns | Low risk | No concerns | No concerns | Some concerns | Major concerns | Very low | ["Within-study bias","Heterogeneity","Incoherence"] |
| 07:09 | 3 | Major concerns | Low risk | No concerns | Some concerns | No concerns | No concerns | Low | ["Within-study bias","Imprecision"] |
| 08:09 | 3 | Major concerns | Low risk | No concerns | No concerns | Some concerns | Major concerns | Very low | ["Within-study bias","Heterogeneity","Incoherence"] |
| 06:10 | 2 | Major concerns | Low risk | No concerns | No concerns | Some concerns | No concerns | Low | ["Within-study bias","Heterogeneity"] |
| 07:10 | 2 | Major concerns | Low risk | No concerns | Some concerns | No concerns | No concerns | Low | ["Within-study bias","Imprecision"] |
| 08:10 | 2 | Major concerns | Low risk | No concerns | No concerns | Major concerns | No concerns | Low | ["Within-study bias","Heterogeneity"] |
| 09:10 | 2 | Major concerns | Low risk | No concerns | No concerns | Major concerns | No concerns | Low | ["Within-study bias","Heterogeneity"] |
| 10:11 | 2 | Major concerns | Low risk | No concerns | No concerns | Major concerns | Major concerns | Very low | ["Within-study bias","Heterogeneity","Incoherence"] |
| 10:12 | 2 | Major concerns | Low risk | No concerns | Some concerns | No concerns | Major concerns | Very low | ["Within-study bias","Imprecision","Incoherence"] |
| 10:13 | 2 | Major concerns | Low risk | No concerns | Some concerns | No concerns | Major concerns | Very low | ["Within-study bias","Imprecision","Incoherence"] |
| 10:14 | 2 | Major concerns | Low risk | No concerns | Some concerns | No concerns | Major concerns | Very low | ["Within-study bias","Imprecision","Incoherence"] |
| 06:11 | 2 | Major concerns | Low risk | No concerns | No concerns | Some concerns | No concerns | Low | ["Within-study bias","Heterogeneity"] |
| 07:11 | 2 | Major concerns | Low risk | No concerns | No concerns | Some concerns | No concerns | Low | ["Within-study bias","Heterogeneity"] |
| 08:11 | 2 | Major concerns | Low risk | No concerns | Some concerns | No concerns | No concerns | Low | ["Within-study bias","Imprecision"] |
| 09:11 | 2 | Major concerns | Low risk | No concerns | No concerns | Major concerns | No concerns | Low | ["Within-study bias","Heterogeneity"] |
| 11:12 | 2 | Major concerns | Low risk | No concerns | No concerns | Major concerns | Major concerns | Very low | ["Within-study bias","Heterogeneity","Incoherence"] |
| 11:13 | 2 | Major concerns | Low risk | No concerns | Some concerns | No concerns | Major concerns | Very low | ["Within-study bias","Imprecision","Incoherence"] |
| 11:14 | 2 | Major concerns | Low risk | No concerns | Some concerns | Some concerns | Major concerns | Very low | ["Within-study bias","Imprecision","Heterogeneity","Incoherence"] |
| 06:12 | 2 | Major concerns | Low risk | No concerns | No concerns | No concerns | No concerns | Moderate | ["Within-study bias"] |
| 07:12 | 2 | Major concerns | Low risk | No concerns | No concerns | Some concerns | No concerns | Low | ["Within-study bias","Heterogeneity"] |
| 08:12 | 2 | Major concerns | Low risk | No concerns | Some concerns | No concerns | No concerns | Low | ["Within-study bias","Imprecision"] |
| 09:12 | 2 | Major concerns | Low risk | No concerns | Some concerns | No concerns | No concerns | Low | ["Within-study bias","Imprecision"] |
| 12:13 | 2 | Major concerns | Low risk | No concerns | No concerns | Major concerns | Major concerns | Very low | ["Within-study bias","Heterogeneity","Incoherence"] |
| 12:14 | 2 | Major concerns | Low risk | No concerns | No concerns | Major concerns | Major concerns | Very low | ["Within-study bias","Heterogeneity","Incoherence"] |
| 06:13 | 2 | Major concerns | Low risk | No concerns | No concerns | No concerns | No concerns | Moderate | ["Within-study bias"] |
| 07:13 | 2 | Major concerns | Low risk | No concerns | No concerns | Some concerns | No concerns | Low | ["Within-study bias","Heterogeneity"] |
| 08:13 | 2 | Major concerns | Low risk | No concerns | No concerns | Some concerns | No concerns | Low | ["Within-study bias","Heterogeneity"] |
| 09:13 | 2 | Major concerns | Low risk | No concerns | Some concerns | No concerns | No concerns | Low | ["Within-study bias","Imprecision"] |
| 13:14 | 2 | Major concerns | Low risk | No concerns | No concerns | Major concerns | Major concerns | Very low | ["Within-study bias","Heterogeneity","Incoherence"] |
| 06:14 | 2 | Major concerns | Low risk | No concerns | No concerns | No concerns | No concerns | Moderate | ["Within-study bias"] |
| 07:14 | 2 | Major concerns | Low risk | No concerns | No concerns | Some concerns | No concerns | Low | ["Within-study bias","Heterogeneity"] |
| 08:14 | 2 | Major concerns | Low risk | No concerns | Some concerns | No concerns | No concerns | Low | ["Within-study bias","Imprecision"] |
| 09:14 | 2 | Major concerns | Low risk | No concerns | Some concerns | No concerns | No concerns | Low | ["Within-study bias","Imprecision"] |
| 03:06 | 0 | Some concerns | Low risk | No concerns | No concerns | Major concerns | Major concerns | Very low | ["Within-study bias","Heterogeneity","Incoherence"] |
| 03:07 | 0 | Some concerns | Low risk | No concerns | Some concerns | No concerns | Major concerns | Very low | ["Within-study bias","Imprecision","Incoherence"] |
| 03:08 | 0 | Some concerns | Low risk | No concerns | Some concerns | No concerns | Major concerns | Very low | ["Within-study bias","Imprecision","Incoherence"] |
| 03:09 | 0 | Some concerns | Low risk | No concerns | No concerns | Some concerns | Major concerns | Very low | ["Within-study bias","Heterogeneity","Incoherence"] |
| 03:10 | 0 | Some concerns | Low risk | No concerns | No concerns | Some concerns | Major concerns | Very low | ["Within-study bias","Heterogeneity","Incoherence"] |
| 03:11 | 0 | Some concerns | Low risk | No concerns | No concerns | Some concerns | Major concerns | Very low | ["Within-study bias","Heterogeneity","Incoherence"] |
| 03:12 | 0 | Some concerns | Low risk | No concerns | No concerns | Some concerns | Major concerns | Very low | ["Within-study bias","Heterogeneity","Incoherence"] |
| 03:13 | 0 | Some concerns | Low risk | No concerns | No concerns | No concerns | Major concerns | Low | ["Within-study bias","Incoherence"] |
| 03:14 | 0 | Some concerns | Low risk | No concerns | No concerns | Some concerns | Major concerns | Very low | ["Within-study bias","Heterogeneity","Incoherence"] |
| 04:10 | 0 | Major concerns | Low risk | No concerns | No concerns | Some concerns | Major concerns | Very low | ["Within-study bias","Heterogeneity","Incoherence"] |
| 04:11 | 0 | Major concerns | Low risk | No concerns | No concerns | Some concerns | Major concerns | Very low | ["Within-study bias","Heterogeneity","Incoherence"] |
| 04:12 | 0 | Major concerns | Low risk | No concerns | No concerns | No concerns | Major concerns | Low | ["Within-study bias","Incoherence"] |
| 04:13 | 0 | Major concerns | Low risk | No concerns | No concerns | No concerns | Major concerns | Low | ["Within-study bias","Incoherence"] |
| 04:14 | 0 | Major concerns | Low risk | No concerns | No concerns | No concerns | Major concerns | Low | ["Within-study bias","Incoherence"] |
| 05:10 | 0 | Major concerns | Low risk | No concerns | No concerns | Some concerns | Major concerns | Very low | ["Within-study bias","Heterogeneity","Incoherence"] |
| 05:11 | 0 | Major concerns | Low risk | No concerns | No concerns | No concerns | Major concerns | Low | ["Within-study bias","Incoherence"] |
| 05:12 | 0 | Major concerns | Low risk | No concerns | No concerns | No concerns | Major concerns | Low | ["Within-study bias","Incoherence"] |
| 05:13 | 0 | Major concerns | Low risk | No concerns | No concerns | No concerns | Major concerns | Low | ["Within-study bias","Incoherence"] |
| 05:14 | 0 | Major concerns | Low risk | No concerns | No concerns | No concerns | Major concerns | Low | ["Within-study bias","Incoherence"] |

D) Certainty of the evidence of the second subnetwork for EC condition.

| **Comparison** | **Number of studies** | **Within-study bias** | **Reporting bias** | **Indirectness** | **Imprecision** | **Heterogeneity** | **Incoherence** | **Confidence rating** | **Reason(s) for downgrading** |
| --- | --- | --- | --- | --- | --- | --- | --- | --- | --- |
| 03:04 | 5 | No concerns | Low risk | No concerns | No concerns | No concerns | Some concerns | Moderate | ["Incoherence"] |
| 03:05 | 5 | No concerns | Low risk | No concerns | No concerns | No concerns | Some concerns | Moderate | ["Incoherence"] |
| 04:05 | 7 | Some concerns | Low risk | No concerns | No concerns | No concerns | Major concerns | Low | ["Within-study bias","Incoherence"] |
| 04:06 | 1 | Major concerns | Low risk | No concerns | No concerns | No concerns | Some concerns | Low | ["Within-study bias","Incoherence"] |
| 04:07 | 1 | Major concerns | Low risk | No concerns | No concerns | No concerns | No concerns | Moderate | ["Within-study bias"] |
| 04:08 | 1 | Major concerns | Low risk | No concerns | No concerns | No concerns | No concerns | Moderate | ["Within-study bias"] |
| 04:09 | 1 | Major concerns | Low risk | No concerns | No concerns | No concerns | No concerns | Moderate | ["Within-study bias"] |
| 05:06 | 1 | Major concerns | Low risk | No concerns | No concerns | Some concerns | No concerns | Low | ["Within-study bias","Heterogeneity"] |
| 05:07 | 1 | Major concerns | Low risk | No concerns | No concerns | No concerns | Some concerns | Low | ["Within-study bias","Incoherence"] |
| 05:08 | 1 | Major concerns | Low risk | No concerns | No concerns | No concerns | No concerns | Moderate | ["Within-study bias"] |
| 05:09 | 1 | Major concerns | Low risk | No concerns | No concerns | No concerns | No concerns | Moderate | ["Within-study bias"] |
| 06:07 | 3 | Major concerns | Low risk | No concerns | No concerns | No concerns | No concerns | Moderate | ["Within-study bias"] |
| 06:08 | 3 | Major concerns | Low risk | No concerns | No concerns | Some concerns | No concerns | Low | ["Within-study bias","Heterogeneity"] |
| 06:09 | 3 | Major concerns | Low risk | No concerns | No concerns | No concerns | No concerns | Moderate | ["Within-study bias"] |
| 07:08 | 3 | Major concerns | Low risk | No concerns | No concerns | No concerns | No concerns | Moderate | ["Within-study bias"] |
| 07:09 | 3 | Major concerns | Low risk | No concerns | No concerns | No concerns | No concerns | Moderate | ["Within-study bias"] |
| 08:09 | 3 | Major concerns | Low risk | No concerns | No concerns | No concerns | No concerns | Moderate | ["Within-study bias"] |
| 06:10 | 2 | Major concerns | Low risk | No concerns | No concerns | No concerns | No concerns | Moderate | ["Within-study bias"] |
| 07:10 | 2 | Major concerns | Low risk | No concerns | No concerns | No concerns | No concerns | Moderate | ["Within-study bias"] |
| 08:10 | 2 | Major concerns | Low risk | No concerns | No concerns | No concerns | No concerns | Moderate | ["Within-study bias"] |
| 09:10 | 2 | Major concerns | Low risk | No concerns | No concerns | No concerns | No concerns | Moderate | ["Within-study bias"] |
| 10:11 | 2 | Major concerns | Low risk | No concerns | No concerns | No concerns | Major concerns | Low | ["Within-study bias","Incoherence"] |
| 10:12 | 2 | Major concerns | Low risk | No concerns | No concerns | No concerns | Major concerns | Low | ["Within-study bias","Incoherence"] |
| 10:13 | 2 | Major concerns | Low risk | No concerns | No concerns | No concerns | Major concerns | Low | ["Within-study bias","Incoherence"] |
| 10:14 | 2 | Major concerns | Low risk | No concerns | No concerns | Some concerns | Major concerns | Very low | ["Within-study bias","Heterogeneity","Incoherence"] |
| 06:11 | 2 | Major concerns | Low risk | No concerns | No concerns | No concerns | No concerns | Moderate | ["Within-study bias"] |
| 07:11 | 2 | Major concerns | Low risk | No concerns | No concerns | Some concerns | No concerns | Low | ["Within-study bias","Heterogeneity"] |
| 08:11 | 2 | Major concerns | Low risk | No concerns | No concerns | No concerns | No concerns | Moderate | ["Within-study bias"] |
| 09:11 | 2 | Major concerns | Low risk | No concerns | No concerns | No concerns | No concerns | Moderate | ["Within-study bias"] |
| 11:12 | 2 | Major concerns | Low risk | No concerns | No concerns | No concerns | Major concerns | Low | ["Within-study bias","Incoherence"] |
| 11:13 | 2 | Major concerns | Low risk | No concerns | No concerns | No concerns | Major concerns | Low | ["Within-study bias","Incoherence"] |
| 11:14 | 2 | Major concerns | Low risk | No concerns | No concerns | No concerns | Major concerns | Low | ["Within-study bias","Incoherence"] |
| 06:12 | 2 | Major concerns | Low risk | No concerns | No concerns | No concerns | No concerns | Moderate | ["Within-study bias"] |
| 07:12 | 2 | Major concerns | Low risk | No concerns | No concerns | Some concerns | No concerns | Low | ["Within-study bias","Heterogeneity"] |
| 08:12 | 2 | Major concerns | Low risk | No concerns | No concerns | No concerns | No concerns | Moderate | ["Within-study bias"] |
| 09:12 | 2 | Major concerns | Low risk | No concerns | No concerns | No concerns | No concerns | Moderate | ["Within-study bias"] |
| 12:13 | 2 | Major concerns | Low risk | No concerns | No concerns | No concerns | Major concerns | Low | ["Within-study bias","Incoherence"] |
| 12:14 | 2 | Major concerns | Low risk | No concerns | No concerns | No concerns | Major concerns | Low | ["Within-study bias","Incoherence"] |
| 06:13 | 2 | Major concerns | Low risk | No concerns | No concerns | No concerns | No concerns | Moderate | ["Within-study bias"] |
| 07:13 | 2 | Major concerns | Low risk | No concerns | No concerns | Some concerns | No concerns | Low | ["Within-study bias","Heterogeneity"] |
| 08:13 | 2 | Major concerns | Low risk | No concerns | No concerns | No concerns | No concerns | Moderate | ["Within-study bias"] |
| 09:13 | 2 | Major concerns | Low risk | No concerns | No concerns | No concerns | No concerns | Moderate | ["Within-study bias"] |
| 13:14 | 2 | Major concerns | Low risk | No concerns | No concerns | No concerns | Major concerns | Low | ["Within-study bias","Incoherence"] |
| 06:14 | 2 | Major concerns | Low risk | No concerns | No concerns | No concerns | No concerns | Moderate | ["Within-study bias"] |
| 07:14 | 2 | Major concerns | Low risk | No concerns | No concerns | No concerns | No concerns | Moderate | ["Within-study bias"] |
| 08:14 | 2 | Major concerns | Low risk | No concerns | No concerns | Some concerns | No concerns | Low | ["Within-study bias","Heterogeneity"] |
| 09:14 | 2 | Major concerns | Low risk | No concerns | No concerns | Some concerns | No concerns | Low | ["Within-study bias","Heterogeneity"] |
| 03:06 | 0 | Some concerns | Low risk | No concerns | No concerns | No concerns | Major concerns | Low | ["Within-study bias","Incoherence"] |
| 03:07 | 0 | Some concerns | Low risk | No concerns | No concerns | Some concerns | Major concerns | Very low | ["Within-study bias","Heterogeneity","Incoherence"] |
| 03:08 | 0 | Some concerns | Low risk | No concerns | No concerns | No concerns | Major concerns | Low | ["Within-study bias","Incoherence"] |
| 03:09 | 0 | Some concerns | Low risk | No concerns | No concerns | No concerns | Major concerns | Low | ["Within-study bias","Incoherence"] |
| 03:10 | 0 | Some concerns | Low risk | No concerns | No concerns | Some concerns | Major concerns | Very low | ["Within-study bias","Heterogeneity","Incoherence"] |
| 03:11 | 0 | Some concerns | Low risk | No concerns | No concerns | No concerns | Major concerns | Low | ["Within-study bias","Incoherence"] |
| 03:12 | 0 | Some concerns | Low risk | No concerns | No concerns | No concerns | Major concerns | Low | ["Within-study bias","Incoherence"] |
| 03:13 | 0 | Some concerns | Low risk | No concerns | No concerns | No concerns | Major concerns | Low | ["Within-study bias","Incoherence"] |
| 03:14 | 0 | Some concerns | Low risk | No concerns | No concerns | No concerns | Major concerns | Low | ["Within-study bias","Incoherence"] |
| 04:10 | 0 | Major concerns | Low risk | No concerns | No concerns | No concerns | Major concerns | Low | ["Within-study bias","Incoherence"] |
| 04:11 | 0 | Major concerns | Low risk | No concerns | No concerns | No concerns | Major concerns | Low | ["Within-study bias","Incoherence"] |
| 04:12 | 0 | Major concerns | Low risk | No concerns | No concerns | No concerns | Major concerns | Low | ["Within-study bias","Incoherence"] |
| 04:13 | 0 | Major concerns | Low risk | No concerns | No concerns | No concerns | Major concerns | Low | ["Within-study bias","Incoherence"] |
| 04:14 | 0 | Major concerns | Low risk | No concerns | No concerns | No concerns | Major concerns | Low | ["Within-study bias","Incoherence"] |
| 05:10 | 0 | Major concerns | Low risk | No concerns | No concerns | No concerns | Major concerns | Low | ["Within-study bias","Incoherence"] |
| 05:11 | 0 | Major concerns | Low risk | No concerns | No concerns | No concerns | Major concerns | Low | ["Within-study bias","Incoherence"] |
| 05:12 | 0 | Major concerns | Low risk | No concerns | No concerns | No concerns | Major concerns | Low | ["Within-study bias","Incoherence"] |
| 05:13 | 0 | Major concerns | Low risk | No concerns | No concerns | No concerns | Major concerns | Low | ["Within-study bias","Incoherence"] |
| 05:14 | 0 | Major concerns | Low risk | No concerns | No concerns | No concerns | Major concerns | Low | ["Within-study bias","Incoherence"] |
